# Source localization of epileptic spikes using Multiple Sparse Priors

**DOI:** 10.1016/j.clinph.2020.10.030

**Published:** 2020-12-03

**Authors:** Mariano Fernandez-Corazza, Rui Feng, Chengxin Ma, Jie Hu, Li Pan, Phan Luu, Don Tucker

**Affiliations:** a LEICI Instituto de Investigaciones en Electrónica, Control y Procesamiento de Señales, Universidad Nacional de La Plata – CONICET, Argentina; b Department of Neurosurgery, Huashan Hospital of Fudan University, Shanghai, China; c Brain Electrophysiology Laboratory (BEL) Company, Eugene, OR, USA; d NeuroInformatics Center, University of Oregon, Eugene, OR, USA

**Keywords:** Electroencephalography (EEG), Epilepsy, Source localization, Bayesian, Multiple sparse priors, sLORETA

## Abstract

**Objective:**

To evaluate epileptic source estimation using multiple sparse priors (MSP) inverse method and high-resolution, individual electrical head models.

**Methods:**

Accurate source localization is dependent on accurate electrical head models and appropriate inverse solvers. Using high-resolution, individual electrical head models in fifteen epilepsy patients, with surgical resection and clinical outcome as criteria for accuracy, performance of MSP method was compared against standardized low-resolution brain electromagnetic tomography (sLORETA) and coherent maximum entropy on the mean (cMEM) methods.

**Results:**

The MSP method performed similarly to the sLORETA method and slightly better than the cMEM method in terms of success rate. The MSP and cMEM methods were more focal than sLORETA with the advantage of not requiring an arbitrary selection of a hyperparameter or thresholding of reconstructed current density values to determine focus. MSP and cMEM methods were better than sLORETA in terms of spatial dispersion.

**Conclusions:**

Results suggest that the three methods are complementary and could be used together. In practice, the MSP method will be easier to use and interpret compared to sLORETA, and slightly more accurate and faster than the cMEM method.

**Significance:**

Source localization of interictal spikes from dense-array electroencephalography data has been shown to be a reliable marker of epileptic foci and useful for pre-surgical planning. The advantages of MSP make it a useful complement to other inverse solvers in clinical practice.

## 1. Introduction

Epilepsy is a neurological disorder of recurring seizures that affects approximately 0.5 to 1 % of the world population (Sander, 2003). These seizures reflect abnormally synchronized neural activity (Blume et al., 2002). The ictal (seizure) oscillations and interictal spikes of epilepsy can be observed in the electroencephalogram (EEG), a recording of biopotentials at the head surface. The list of diseases associated with epilepsy and thus, the possible causes of epilepsy is vast (Engel, 2001). Some epilepsy cases are correlated with brain lesions or abnormalities, visible by neuroimaging techniques such as computed tomography (CT) or magnetic resonance imaging (MRI), while others are not associated with a visible lesion. About 30% of epileptic patients are non-responsive to drug treatment and neurosurgical resection of the epileptogenic tissue becomes the primary therapeutic option. The goal in pre-surgical planning is to determine the location of the so called “epileptogenic zone”, theoretically defined as the smallest brain region that, if removed or disconnected, will result in the patient being seizure free (Lüders et al., 2006). More recently, the concept of “epilepsy networks” has gained popularity, where a treatment directed at any region of the network should, conceptually, be just as effective as treatments directed at a specific ‘focus’ of seizure activity (Jehi, 2018; Spencer, 2002).

Virtual head models are built to compute what is called the *forward problem* in EEG, i.e., the computation, governed by the well-known Maxwell equations, of the expected scalp measurements produced by a source of neuronal activity. This computation produces a lead field (LF) matrix that describes the scalp voltage field for neuronal activity at every position on the cortex (i.e., possible EEG sources). The LF matrix is used by inverse solvers to estimate the brain activity that produces the real EEG signal, known as the *inverse problem*. It is well established that accurate LF matrices produce more accurate source estimates (Brodbeck et al., 2011; Feng et al., 2018), and the quality of LF matrices is dependent on the accuracy of head conductivity models used. Geometrically realistic head models with three to eight different tissues require numerical algorithms to solve the forward problem. These algorithms include the boundary element method (de Munck et al., 2000), the finite difference method (Hallez et al., 2007) or the finite element method (FEM) (Beltrachini et al., 2011). Among the realistic head-shaped models, many of them assume nested layers (as required for the boundary element method), and more recent models incorporate multiple skull compartments and foramina (Fernandez-Corazza et al., 2018), the dura layer (Ramon et al., 2006), anisotropic conductivity from diffusion tensor imaging (Wolters et al., 2006) and even major vessels (Fiederer et al., 2016).

Multiple inverse solvers have been proposed in the literature to image the underlying neuronal activity that generates the EEG signals (Grech et al., 2008). The simplest inverse solver is the minimum norm (MN) where the solution is the activity map that minimizes the squared difference between the estimated signals and actual measurements (Hämäläinen and Ilmoniemi, 1994). As the mathematical problem is undetermined, there are infinite possible solutions and thus some sort of regularization is required. The low-resolution electrical tomography (LORETA) method imposes a smoothness constraint (minimum 3D Laplacian) on the solution space (Pascual-Marqui et al., 1994). The standardized low-resolution electrical tomography (sLORETA) is probably the most commonly used method (Pascual-Marqui, 2002). It is simple, fast and, in contrast with MN and LORETA, is unbiased towards the electrode positions.

Bayesian inverse solvers have garnered extensive interest in the past decade. These solvers belong to a separate class and share the common feature that the EEG or magnetoencephalography (MEG) signals being modeled are based on a set of priors (i.e., prior assumptions). A likelihood function is optimized to parametrically estimate the most likely combination of priors. The multiple sparse priors (MSP) method belongs to this family and uses a large set of priors, where the weight for each prior is automatically determined by an expectation maximization algorithm (Friston et al., 2008). The MSP method appears to be robust and accurate, and its performance is improved only slightly by the addition of information, such as localization priors from functional MRI, that have been shown to improve accuracy obtained with other inverse solvers (Daunizeau et al., 2010; Henson et al., 2010). Two extensions of the MSP method were proposed: temporally adaptive - MSP (taMSP) and temporally adaptive with fused lasso and connectivity measure (taMSP-FL-conn) (Martinez-Vargas et al., 2017). These researchers evaluated the algorithms with epilepsy data (two patients, two seizures each) and concluded that their proposed variants were accurate for localizing both focal and distributed sources. Another Bayesian variant and extension of MSP, coherent maximum entropy on the mean (cMEM) method, has been shown, also with a boundary element model, to be accurate for estimating the source of epileptic EEG and MEG data (Chowdhury et al., 2016; Grova et al., 2006). Using data from 15 epileptic patients, cMEM solutions were compared against Bayesian equivalents of MN and LORETA solutions and concludinging, similar to (Friston et al., 2008), that the cMEM method was superior when source estimates were evaluated relative to data obtained from intracranial EEG (icEEG) recordings (Heers et al., 2016). However, this work did not compare cMEM versus MSP.

Thus, evidence is accumulating that the Bayesian approach to source estimation, particularly the MSP method, appears to be superior to other classes of inverse solvers. There are also usability advantages of the MSP method. First, the MSP method does not require selection of additional parameters (e.g., noise handling hyperparameters in MN and sLORETA). Solutions acquired with methods that depend on noise handling hyperparameters will differ depending on hyperparameter choice, and making such choices is challenging for physicians without extensive training and experience. Moreover, MSP solutions are easily interpretable because they are sparse (focal), requiring no magnitude thresholding to reveal location(s). Surprisingly, given the strengths of the MSP method, there has been no study that directly evaluated the basic MSP algorithm for localization of epilepsy activity, either with EEG or MEG data.

In this work, we evaluate our own implementation of the MSP algorithm in combination with our own hexahedral FEM (Hexa-FEM) solutions for head conductivity models. This evaluation is performed using inter-ictal spike data from fifteen patients who also had surgical resection and became seizure-free post-surgery. Importantly, in these 15 patients, the epileptic foci include those in the medial temporal lobe as well as those involving other cortical regions (i.e., extratemporal). The questions we address are: (1) what is the expected performance of the MSP method for the specific application of source localizing epilepsy spikes in real cases?, and (2) how does its performance compare against other methods, such as sLORETA and the most advanced cMEM? Finally, we include a conductivity analysis to understand the impacts of using literature values for the scalp and skull conductivity values.

## 2. Methods

### 2.1. Patients

Fifteen patients undergoing epilepsy pre-surgical evaluation were recruited for the study and provided informed consent (protocol approved by the Institution Review Board at Fudan University). In addition to dense-array EEG (dEEG) assessment, patients had standard pre-surgical workup, which included long-term 16-channel video EEG, semiology evaluation, Fluoro-2-deoxy-D-glucose positron emission tomography (FDG-PET) and MRI. Half of the subjects were evaluated also with icEEG recordings before surgery. Patient information is presented in Table 1.

### 2.2. High-resolution electrical head models

To create high-resolution individual head models, all patients had axial T1-weighed MRI scans, acquired using the 3D-SPGR sequence (General Electric, US) with 1x1x1 mm resolution and scanned from top of head to chin. The MRI data were then segmented with the GeoSource 3.0 software (Philips Neuro, Eugene, Oregon, USA), which uses a unique relative thresholding method for tissue segmentation (Li et al., 2016). Tissue segmentation classified each image voxel into the following tissue types: eyeball, scalp, skull, cerebral-spinal fluid (CSF), gray matter (GM), white matter (WM) and air. The WM and GM were further partitioned into cortex and cerebellum.

To specify current generator positions (i.e., dipole sources), the cortical surface was first characterized using triangular meshes, which were parceled into patches of approximately equal size. All models used in the present study contained 2400 dipole patches per hemisphere. For each patch, perpendicular directions of vertices within the patch were averaged to derive the average, perpendicular orientation of current flow. Average sensor positions were used to register to each head model.

There are several options to solve the differential equations involved in the FP. These numerical methods transform the analytic solutions into a linear system of equations. The finite difference method is one option that runs directly on the segmented voxel space, and it is more computationally intensive than other methods (Turovets et al., 2014). Another option is the use of the boundary element method, which is computationally efficient but requires the inaccurate assumption that major tissues such as the skull form closed boundaries (misrepresenting major skull orifices such as foramen magnum or optical canals). A different method is the tetrahedral FEM. It has the advantage that smooth surfaces can be built because the meshing process is adaptive and is not necessarily tied to the original MRI voxel space. Also, coarser or finer regions can be defined according to the desired precision.

In the present work we used HexaFEM. HexaFEM has been widely used for mechanical simulations, although not so commonly used for electromagnetic head modelling. The main advantage is that the mesh can be built directly from the segmented image where each voxel is directly mapped as a cube of the mesh, no matter the complexity of the segmentation. HexaFEM also has the advantages of tetrahedral FEM in that the resulting matrices are both sparse and symmetric, allowing the use of computationally efficient solvers to solve the resulting linear system of equations. Also, it has the potential of being adaptive, although for the current version we kept it as one cube per voxel. In Appendix A, we show a comparison of HexaFEM against analytics and the finite difference method in a three-layer spherical model, and against the finite difference method in a realistic model. The EEG forward problem computation for each dipole is intensive. The time to compute the LF matrix can be drastically reduced if the reciprocity principle is used (Malmivuo and Plonsey, 1995). It relates the EEG forward problem with the transcranial electrical stimulation (TES) forward problem that computes the electric field at the brain from a current injection pattern. In this way, instead of the larger number of dipole computations, only the smaller number of electrode computations are required.

We assigned literature conductivity values for each tissue as follows: 0.35S/m for the WM (Gabriel et al., 1996), 0.01 S/m for the skull (Oostendorp et al., 2000), 0.33 S/m for the scalp (Goncalves et al., 2003), 1.79S/m for the CSF (Baumann et al., 1997), 0.25 for the GM (Gabriel et al., 1996) and 1.55 S/m for the eyeballs (Lindenblatt and Silny, 2001).

### 2.3. Dense-array EEG acquisition

dEEG data were acquired in one session, lasting approximately one hour for each patient. Patients were allowed to rest in a recumbent position during the recording session. dEEG were acquired using 256-channel system (Philips Neuro, Eugene, Oregon, USA). Each EEG sensor net was applied using the nasion, bilateral pre-auricular locations and Cz position as landmarks to ensure standardized placement across patients. The tension structure of the EEG net ensured even distribution of the remaining sensors on the head and at similar locations across patients. The EEG was recorded with a direct current amplifier and sampled at 500 or 1000 s/s.

### 2.4. Inter-ictal spike marking

The continuous dEEG data for each patient were filtered with a 0.1–30 Hz band-pass filter. The data were then reviewed by a member of the epilepsy evaluation team (R. F.) and interictal spike types marked. Topographic maps, after the data were referenced to the average-reference, were used to examine spatial distribution, and spikes were grouped according to spatial similarity prior to spike averaging. Spike dominance was determined based on frequency of occurrence. The average dominant spike was used in source estimation. Spikes were segmented at the peak of the spike and included 500 ms before and after spike peak. Based on previous comparisons to intracranial validations, source estimates were based on the rising slope prior to the spike peak. Bad channels, after averaging, were manually identified and removed prior to source estimation.

### 2.5. Specifying surgically resected brain region

**Fig. 1.**
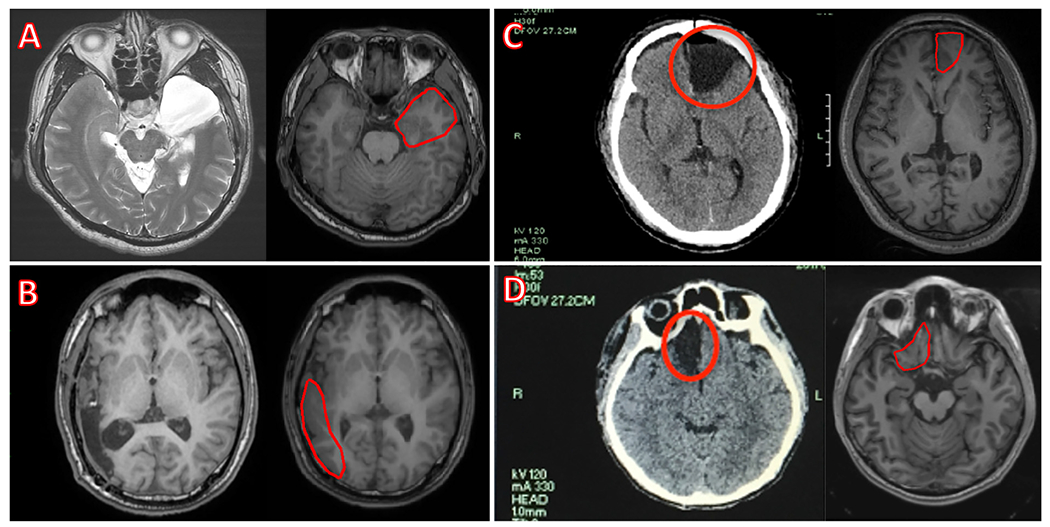
Examples of the volume of interest (VOI) marking process. VOIs marked on the pre-surgical magnetic resonance image (MRI) (the right figure of each pair of images) based on the post-surgical MRIs for (A) subject S5 and (B) subject S9; or on the post-surgical CT images for (C) subject S7 and (D) subject S6 are shown.

For each patient, the volume of the resected region was marked by hand on the pre-surgical MRI by the neurosurgeon that performed the resection. The neurosurgeon used all available information such as post-surgical MRI slices, post-surgical CT images, brain pictures taken during the surgeries, pre-surgical PET and presurgical intracranial EEG data as guidelines while marking the resected region in the pre-surgical MRI. Use of the pre-surgical MRI instead of the post-resection MRI was adopted for several reasons: (i) post-surgical MRI may contain significant brain shifts due to the resection; (ii) the EEG data was collected before the surgery; and (iii) in the majority of the cases, either the post-surgical MRI available for these data was acquired for only few slices of interest and not for the whole brain, or the post-surgical protocol specified acquisition of only CT images. Due to reasons (i) and (ii), using the pre-surgical MRI was preferred even in the case of having the full head post-surgery MRI. Fig. 1 shows examples of the pre-surgical MRIs and the marked volume of interest (VOI) masks overlaid with the corresponding post-surgical MRIs. The volumes were delineated directly on the T1 images using the MIPAV software (https://mipav.cit.nih.gov).

### 2.6. Inverse methods

#### 2.6.1. sLORETA

sLORETA is one of the most popular inverse solvers due to its algorithmic simplicity and unbiased nature (Pascual-Marqui, 2002; Sekihara and Nagarajan, 2008). The formulation is:

(1) $$s\left(r\right)=w\left(r\right)Y=\frac{l{\left(r\right)}^{T}{\left({\mathrm{LL}}^{T}+\alpha I\right)}^{-1}Y}{\sqrt{l{\left(r\right)}^{T}{\left({\mathrm{LL}}^{T}+\alpha I\right)}^{-1}l\left(r\right)}}$$

Where **Y** is the *L* × *T* EEG data matrix (where *L* is the number of channels and *T* is the number of time samples), **L** is the LF matrix, **l**(*r*) is the column of the LF matrix generated by the dipolar source *r*, α is the hyperparameter, **I** is the identity matrix, **s**(*r*) is the source waveform estimate of dipole *r*, and **w**(*r*) is the weighting vector for dipole *r*. Formulation in Eq. (1) is repeated for each dipole *r* of a total of *D* dipoles.

#### 2.6.2. Multiple sparse priors (MSP)

The MSP method is described in detail in the original paper (Friston et al., 2008). This method defines a large set of sparse priors that are later weighted to better explain the measured data. The weighting is done by using an expectation-maximization iterative algorithm. There is a compromise in defining the set of priors; the priors should be general enough to cover a wide solution space such that the solution is not biased to a small subdomain of the solution space. The function to optimize is a maximum likelihood function.

Implementation of MSP method was accomplished with no modifications to the algorithm as proposed by Friston et al (2008), see Appendix B for some more details taken directly from that source. A step-by-step function evaluation was accomplished to ensure the code performed equal to implementation of the MSP method in the SPM Matlab toolbox (Kiebel and Friston, 2004). Both sLORETA and MSP methods will be available within BEL Co’s Sourcerer™ product. Sourcerer™ will be available as a research application. For clinical use, Sourcerer™ will require FDA clearance.

**Fig. 2.**
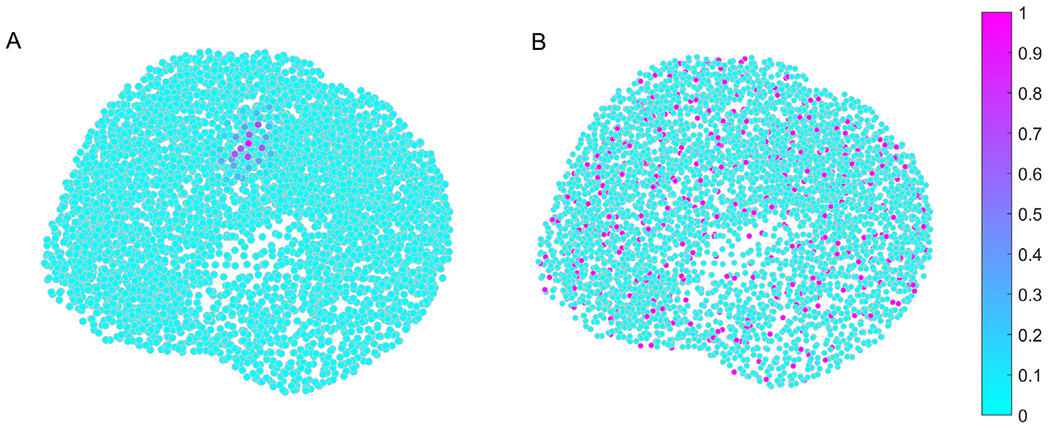
(A) Example of a compact support prior. The color scale indicates the neighbor connection strength, where the strongest one is the central dipole location. (B) Central dipole locations for the 400 priors (in magenta) among all other dipole locations (in cyan). (For interpretation of the references to colour in this figure legend, the reader is referred to the web version of this article.)

The construction of the priors also followed Friston et al (2008) as much as possible. The first step was to generate an adjacency matrix “**A**” (*D* × *D*), with ones indicating neighboring sources and zeros indicating non-neighbors. The neighbors were determined from the cortical surface as neighboring patches and not as the closest patches by Euclidean distance. A spatial smoothing matrix **G**, as $G=\Sigma_{i=0}^{8}\frac{\sigma^{i}}{i!}A^{i}$, was generated, where sigma is a constant (*σ* = 0.6) that weighs the influence of the neighbors, and the number 8 in the sum ensures that up to eight neighbors are engaged. Each column **g**_*i*_ of **G** corresponds to a compact prior with one central element and its neighbors. Fig. 2A shows an example of a prior. A subset of 400 out of 4800 rows **G** was selected as the subset of priors, meaning that approximately 8.5% of the total number of dipoles were selected as the central dipoles of the priors. Fig. 2B highlights the central dipoles of the 400 selected priors, showing that they are evenly distributed in the brain space. Finally, the 400 priors were built as $Q_{i}=g_{i}g_{i}^{T}$.

Once the hyperparameters corresponding to each prior were computed using the expectation maximization method, the maximum a posteriori estimator was finally computed as:

(2) $$M=\Sigma^{\epsilon }L^{\text{T}}\Sigma^{-1}U^{\text{T}},$$

and the signal estimates in the source space over time were wewe:

(3) $$E_{D\times T}=MY,$$

where Σ^ϵ^ is the estimated spatial covariance matrix in the source space, Σ is the estimated covariance matrix in the electrode space and **U** is the spatial projector of size *L* × *L* accounting for the confounds or fixed effect on the signal model (Friston et al., 2008).

#### 2.6.3. Coherent maximum entropy on the mean (cMEM)

The cMEM method is available in the scientific Matlab toolbox Brainstorm (Tadel et al., 2011). The cMEM method minimizes the Kullback Leibler divergence and incorporates spatial smoothness, similarly to MSP (Chowdhury et al., 2016). The “be_cmem_solver” function from Brainstorm was used for the present study. This function requires the LF matrix and the neighboring matrix **A** (as used in MSP). Neighbors were defined in the same manner as for the MSP method. Algorithmic parameters used default values and the baseline, as required by the method, was defined as the first 100 samples (in all cases the spike starts after these first 100 samples).

### 2.7. Best hyperparameter and polarity selection

For each patient we applied the sLORETA, MSP and cMEM methods and obtained source activity maps. For each algorithm, the following procedure was used to keep the strongest positive, negative or both sources (and the hyperparameter for LORETA) by comparing the true EEG and artificial EEG generated by these sources as follows:

– For sLORETA, 9 source activity maps were computed using 9 different hyperparameters, ranging from 1 × 10^−4^ to 1 × 10^4^, and increasing one order of magnitude at each step. For each solution obtained with each hyperparameter, three candidate dipole selections were considered based on polarity: (i) the two dipoles with most positive intensity (“max”), (ii) the two dipoles with most negative intensity (“min”), and (iii) the dipole with most positive intensity and the dipole with most negative intensity (“both”). Note that a solution being positive or negative reflects the polarity of the source with respect to the cortex normal surface. Thus, a positive sign indicates a source, reflecting current flow outward from the cortex, and a negative sign indicates a sink, reflecting inward current flow. The three options were considered because we observed that in certain cases the polarity of the real EEG data did not correspond to the polarity of the strongest (in module) source. Among the 27 candidates (9 hyperparameter solutions with three possible polarities each), the one that better explained the scalp EEG pattern was chosen. This procedure was done by placing artificial EEG generators (dipoles) centered at each of the 27 possible source locations and simulating the EEG potential at the scalp. Then, this synthetic EEG potential was compared to the actual measurements by using the normalized relative difference measure (NRDM^2^) (Meijs et al., 1989) and the solution with lowest NRDM value was chosen. Note that this search of the best source localization does not require the knowledge of a ground truth.

– For MSP and cMEM methods, because there is no hyperparameter requirement, we followed the same procedure as for sLOR-ETA but just for the three polarities: “min”, “max” or “both”. Again, the solution that better matched the EEG recording on the scalp by means of the NRDM metric was chosen.

The time point to analyze was selected at the spike onset prior to the spike maximum by visual inspection of the EEG measurements over time (butterfly plot) and as well the spatial topography of the voltage data. The same EEG time point was used for the three solvers. It is noted, however, that the solutions were robust across multiple time points of the rising edge.

### 2.8. Evaluation metrics

#### 2.8.1. Center of mass and localization error

The most important metric of success was defined as the Euclidean distance between the center of mass (CoM) of selected dipoles from the solution and the closest VOI voxel. The CoM is defined as:

(4) $$CoM=\frac{\Sigma_{selected\;dipoles}\left|s_{i}\right|\vec{\chi_{i}}}{\Sigma_{selected\;dipoles}\left|s_{i}\right|}$$

where *s_i_* is the intensity of the solution at dipole *i*, and $\vec{\chi_{i}}$ are the spatial coordinates of dipole *i*. The selected dipoles were either the most intense ones with positive polarity, the most intense ones with negative polarity or a combination of both, as described in Section 2.7. The distance of the CoM and the VOI can be interpreted as a localization error (LE) or bias metric. This quality metric assumes that the marked VOI is the ground truth for the location of the spike generators. This assumption has limitations that are discussed in Section 4.3.

#### 2.8.2. Spatial dispersion and focality

These metrics were used to quantify the extent and focality of the reconstruction for the three solutions when the CoM was located inside the VOI or close enough (less than 5 mm). We defined the spatial dispersion (SD) as (Chowdhury et al., 2016):

(5) $$SD=\sqrt{\frac{\Sigma_{all\;dipoles}\left(\underset{j\in VOI}{\min}D_{ij}^{2}\right)s_{i}^{2}}{\Sigma_{all\;dipoles}s_{i}^{2}}}$$

In the numerator of Eq. (5), the squared of each intensity *s_i_* is weighted by the minimum squared distance between source *i* and the VOI (*Dij*). This means that if larger intensities are inside the VOI, they are weighted by zero. Overall, a large SD indicates that there are large intensity spots outside the VOI and the further away they are, the more weight they are given. In contrast, a lower SD means that the source activation map has stronger activity in the VOI or nearby.

Because SD strongly penalizes source activity far away from the VOI, a simpler focality metric (Foc) was also computed and defined as:

(6) $$Foc=\sqrt{\frac{\Sigma_{VOI\;dipoles}S_{i}^{2}}{\Sigma_{all\;dipoles}S_{i}^{2}}}$$

The numerator of Eq. (6) sums the squared of each intensity *s_i_* that is inside the VOI-plus-5 mm and the denominator is equal to Eq. (5). The Foc metric lies in the [0–1] range, where lower values indicate poor focality and values closer to 1 indicate better focality.

## 3. Results

Results obtained using the LE, SD and Foc metrics for each of the algorithms against the hand-marked VOIs are presented first. Results using an alternative VOI definition (based on an atlas parcellation criterion) are presented next. An analysis of the source localization robustness against variations of the electrical conductivity values of the tissues is presented last.

### 3.1. Results based on hand-marked VOI

The LE results for sLORETA, MSP and cMEM methods are shown in Table 2. When the LE is lower than 1 mm, it means that the CoM is inside the VOI. For the sLORETA solutions, the resulting hyperparameters and polarities (min, max or both) are shown, whereas for MSP and cMEM solutions only the resulting polarities are relevant.

We defined a LE below 5 mm as good (in bold in Table 2), because it is close enough to the VOI and, by visual inspection, appears to be a reasonable tolerance limit to the inherent mis-match between the pre-operative MRI and the resected region. We also distinguish between a close no-hit (LE between 5 mm and 15 mm) and a large no-hit (LE larger than 15 mm) with different font styles in Table 2 (blue and red respectively). Using these criteria, sLORETAand MSP displayed 80% (12/15) accuracy whereas cMEM produced 66% (10/15) accuracy.

Visual inspection of each solution provides a better understanding of each case. Figs. 3, 4, and 5 show the solutions for the first three subjects as examples. Figures for the remaining subjects, as well as a detailed, case-by-case analysis are provided as Supplementary Material (Fig. SF1 for subject S4, Fig. SF2 for S5, Fig. SF3 for S6, Fig. SF4 for S7, Fig. SF5 for S8, Fig. SF6 for S9, Fig. SF7 for S10, Fig. SF8 for S11, Fig. SF9 for S12, Fig. SF10 for S13, Fig. SF11 for S14, Fig. SF12 for S15).

**Fig. 6.**
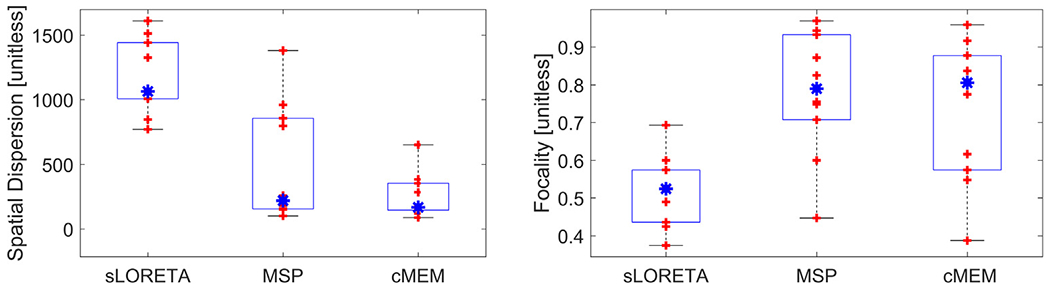
Spatial dispersion and focality. Boxplots of the spatial dispersion (left, the lower the better) and focality (right, the larger the better) of the solutions obtained with the three solvers for the cases where all three algorithms presented a localization error lower than 5 mm (S1, S2, S3, S5, S6, S7, S10, S11, S13, S15). Each red cross represents a different subject, the blue asterisk represents the median, and edges of the blue boxes are the 25th and 75th percentiles. Methods: standardized low-resolution brain electromagnetic tomography (sLORETA), multiple sparse priors (MSP) and coherent maximum entropy on the mean (cMEM). (For interpretation of the references to colour in this figure legend, the reader is referred to the web version of this article.)

Fig. 6 shows the SD and the Foc metrics of the cases where the LE is lower than 5 mm for the three solvers. As expected, sLORETA solutions have larger SD and are less focal than MSP and cMEM solutions. The median of the SD is very similar for MSP and cMEM solutions. However, the MSP method produces solutions that can be more dispersed (S3, S7, S10, S13) than the cMEM solutions. This is because the MSP solutions present, in some cases, attenuated activity far away from the maxima that affects the SD (because they are weighted by a large distance). For example, in Fig. 6 the MSP solution for subject S3 presents some very light activity in the frontal pole and in some other brain areas far from the VOI that the cMEM does not. In terms of focality, where the inside of VOI-plus-5 mm energy is divided by the overall energy without weighting by the distance, the MSP and cMEM solutions are more focal than the sLORETA solutions, and MSP solutions are slightly more focal than cMEM solutions, although again, the median of these two methods is very similar.

The results shown in Table 2 might be slightly influenced by the boundaries of the VOI that were marked by hand by the neurosurgeon. As an example, subject S4 has the smallest VOI and it is the only pediatric subject. The three methods, especially sLORETA and cMEM, seem to give a source localization in the same brain region of the VOI, although in Table 2 the LE is marked as large (more than 15 mm). Thus, in the following section we present alternative results based on a standard parcellation, to address this limitation.

### 3.2. Results based on atlas parcellation

The method used to segment the head and the cortex into dipole patches also provides a transformation map from the native space to Talairach space (Li et al., 2016). Using this map, we obtained the Talairach coordinates of every voxel of the VOI. From these coordinates and using the Bisweb MNI-TAL conversion tool (https://bioimagesuiteweb.github.io/webapp/mni2tal.html) the corresponding Brodmann area (BA) for each voxel was obtained. Through this procedure, a list of the BAs that are “touched” by each VOI was generated. Because some VOIs have only few voxels inside some BAs, only BAs with more than 50 mm^3^ were retained in the list. New boundaries for each VOI in the normalized space were defined as the boundaries of the retained BAs, following the standard atlas parcellation. In this way, the hand-marked VOIs are only used as a rough reference to indicate the major involved BAs, but do not define the new VOI boundaries. Therefore, this method is robust to possible imprecise VOI boundary errors made in the hand-marking process.

The CoMs of each solution were also mapped to the MNI space, and a check was performed to determine whether they fall inside or outside the new definition of VOIs by examining the correspondence between BAs of the VOIs and the BAs of the CoM. If the BA of the CoM is within the list of the BAs involved in the VOI, then it is considered a “hit”, and otherwise it is labelled as a “miss”. Table 3 shows the lists of BAs involved in each VOI and the BAs of each CoM for each solver. This table only shows the subjects where at least one algorithm failed in Table 2, because the other cases that succeed with all three algorithms did not change with the BA - VOI registration (this is expected because both the VOIs and the CoMs are moved using the same transformation). Comparing the results in Tables 3 and 2, the accuracy of sLORETA solutions remained the same (12/15), being successful for exactly the same subjects. MSP solutions showed an improved accuracy of 13/15, due to inclusion of S8, and cMEM solutions also increased in accuracy (13/15) due to the addition of “hits” for subjects S4, S9 and S14. The cMEM source activation maps for these subjects (Supplementary Figures SF1, SF6 and SF11) indeed show that the cMEM localizations are close to the VOIs.

#### 3.2.1. Conductivity robustness

Because literature conductivity values were used to generate the LF matrices, a study was performed to analyze the robustness of the sLORETA and MSP inverse solutions with respect to the skull and scalp electrical conductivity values. For each patient, additional LF matrices were computed by adjusting ±15% the scalp and skull conductivity values, resulting in eight additional forward solutions. MSP and sLORETA inverse source localization were performed using these new LF matrices. This resulted in a 3x3 LE matrix per subject, where the horizontal axis corresponds to −15%, 0% and 15% deviation of the skull conductivity and the vertical axis corresponds to the −15%, 0% and 15% deviations of the scalp conductivity.

**Fig. 7.**
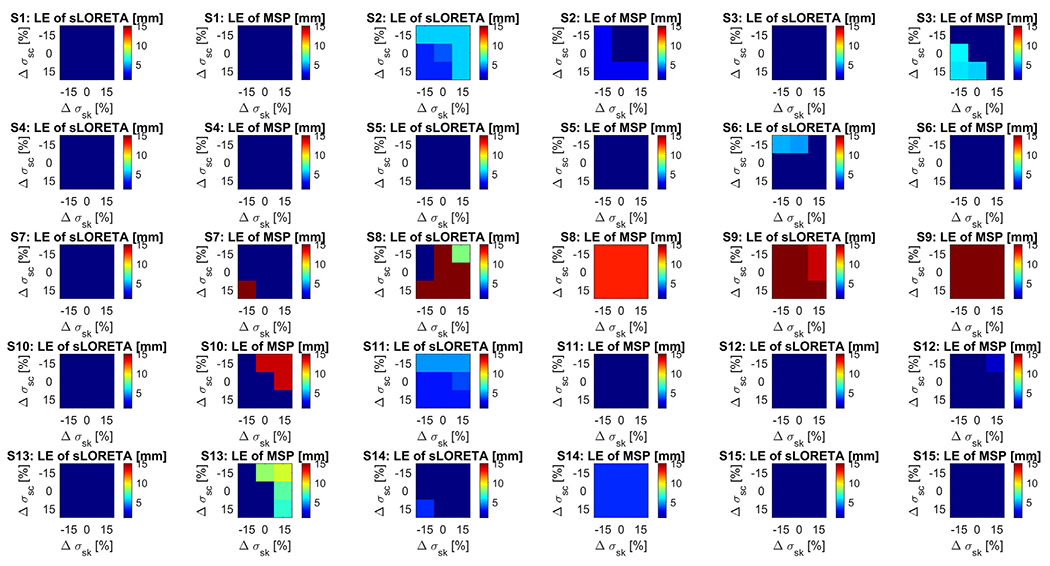
Localization errors (LE) when using deviations of the electrical conductivity. Each chart indicates the LE for each subject and each of the two source localization methods: sLORETA and MSP. Within each chart, the horizontal axis corresponds to −15%, 0% and 15% deviation of the skull conductivity with respect to the nominal (Δ*σ_sk_*) and the vertical axis corresponds to the −15%, 0% and 15% deviations of the scalp conductivity (Δ*σ_sc_*). Note that the central point of each chart indicates the LE using the nominal values for the scalp and skull conductivity values, that correspond to the values shown in Table 2. Methods: standardized low-resolution brain electromagnetic tomography (sLORETA), and multiple sparse priors (MSP). (For interpretation of the references to colour in this figure legend, the reader is referred to the web version of this article.)

Fig. 7 shows the LE matrices for all subjects, where the color indicates the LE. Note that the color scaling is the same for all cases and that a flat color image indicates that the LE did not change when using different electrical conductivity values.

## 4. Discussion

### 4.1. Comparison between sLORETA, MSP and cMEM

In this work we compared the performance of the popular sLORETA and the most advanced MSP and cMEM algorithms to source localize epilepsy spikes, considering the resected brain region as a ground truth VOI. Results show that the three inverse methods were able to localize the epileptic spike onsets to the resected brain region in the majority of cases, even for this set of complex cases with only seven (S1, S5, S8, S10, S11, S12, and S15) out of fifteen subjects being typical temporal lobe epilepsy. sLORETA and MSP methods achieved an accuracy of 80% (12/15), and they failed for the same subjects (S4, S8 and S9), whereas the cMEM method achieved an accuracy of 66% (10/15). However, when it failed, cMEM had an LE lower than 2.5 cm in 4/5 cases. This explains why when analyzing the alternative VOI based on a normalized brain segmentation, cMEM achieved an accuracy of 86%, same as MSP and slightly better than sLORETA (80%).

Considering only the typical anterior temporal lobe cases (S1, S5, S8, S10, S11, S12, S15), accuracy was 86% (6/7) for MSP and sLORETA methods and 70% (5/7) for the cMEM method for hand-segmented VOIs, and 86% (6/7) for sLORETA, 70% (5/7) for cMEM, and 100% for the MSP method for atlas-based VOIs. Excluding the anterior temporal lobe cases, the success rate was 75% (6/8) for sLORETA and MSP methods and 62% (5/8) for the cMEM method for hand-segmented VOIs. For atlas-based VOIs, these rates were 75% (6/8), 75% (6/8) and 100% (7/7) for sLORETA, MSP and cMEM methods, respectively.

The success rates obtained here are in general agreement with what has been reported in a more extensive study with 152 patients that used dEEG and surgical resection plus clinical outcome for ground truth comparisons (Brodbeck et al., 2011). In that study, 52 patients had individual MRIs that were used to construct individual head models. In this subset, the researchers showed accurate localization in 86% of the cases when using the LAURA method as the inverse solver. From those patients, 52% (27/52) had extratemporal spikes, similarly to our set (53%). This study showed that when extratemporal cases were evaluated, the accuracy was 75%, in agreement with our findings.

Even when two or three of the inverse algorithms localized the source within the VOI, sometimes they do not agree on the exact locations. This fact highlights the difference in nature between the three algorithms and supports the idea that they can be complementary, in agreement with (Grova et al., 2006). In seven out of twelve cases where the sLORETA and the MSP methods produced results in agreement with the VOI, these show exact (or almost exact) match (S1, S2, S3, S5, S6, S7, S15). For the other three subjects (S10, S11, S13), the solutions of both methods are very close but at the opposite side of the cortex, with one solution pointing inward and the other outward from the cortical surface. For the remaining 2 subjects (S12 and S14), it seems that the solutions are very close on the wrinkled cortical surface, although they are separated when depicted on the inflated cortical surfaces.

In five out of the 10 cases that the cMEM solutions correspond to the VOI, the strongest cMEM activations agree with the strongest activations of the sLORETA and/or MSP solutions (or its immediate neighbors, see S1, S3, S5, S10, S15), but it does not exactly match in the others cases (S2, S6, S7, S11 and S13). However, in two cases where both sLORETA and MSP solutions fail (S4, S9), the cMEM solution is much closer to the VOI. For subjects S4, S8 and S9, where the three methods fail, one or two of the algorithms are much closer to the VOI than the other(s). However, it is interesting to note that when a method fails, there is always some secondary activity that coincides with the main peak of the algorithm that succeeded. Thus, slight differences in the ways the sources are weighted by each algorithm can make the difference between a hit or a miss. Therefore, a visual inspection of the secondary sources when using multiple algorithms to the same data and head models might help in distinguishing the most important source location.

Visual inspection of the source activity maps clearly shows that the MSP solutions generally have more pronounced “peaks” than the sLORETA and cMEM solutions, which is consistent with the quantitative results showing better focality of the MSP compared to both sLORETA cMEM solutions (the latter to a lesser extent). The MSP maps are easier to interpret and do not require arbitrary thresholds for visualization. The spatial dispersion metric showed that both MSP and cMEM have less dispersion than sLORETA. The blurriness and source extension overestimation of sLORETA are well known (Cosandier-Rimélé et al., 2017; Wagner et al., 2003). If thresholds are not applied in sLORETA, source reconstruction of temporal lobe spikes typically extends to the orbito-frontal region (see subjects S1, S5, S8, S10, S11, S12, S15), in agreement with previous findings (Plummer et al., 2010). In some specific examples, the MSP solutions show secondary peaks far from the most intense source of activity that alter this metric because it significantly penalizes distant activity sources. The cMEM solutions in general show broader activity regions than MSP but are concentrated in only one cluster.

We proposed, for the three methods, a simple algorithm to objectively select the best solution among a range of possible hyperparameters (only for sLORETA) and the polarity type (min, max or both) based on comparing the true EEG and the synthetic EEG generated by the peaks of the solutions. This procedure was inspired by consistency checks that some specialists perform in practice. The accuracy of sLORETA solutions was achieved by testing a wide range of hyperparameters whereas the MSP and cMEM methods automatically adjust the hyperparameters, which can be considered as an advantage over sLORETA. In terms of the similarity between the reconstructed EEG with the three solutions and the true scalp EEG topography, there is no clear trend to determine which of the solutions produce a more similar EEG topography. An important implication of these results is that despite being sparse by nature, the MSP solution can sometimes produce a very similar EEG to the measured EEG, meaning that the true EEG is compatible with only one very focal generator in these cases.

The MSP version used here followed as much as possible the original methodology described in (Friston et al., 2008). However, there are some modifications that can potentially enhance the method. Although the selection of 400 priors out of 4800 seems to be a good compromise between computational time and coverage, the number of priors can be enlarged to have a better coverage of the cortex. Another possibility is to increment the value of parameter *σ* in matrix **G** leading to broader priors. In future studies it would be beneficial to examine accuracy as a function of how these priors are adjusted.

Regarding the cMEM method, the Brainstorm default parameters were used and the adjacency matrix **A** was adopted from that used for MSP method. A difference between cMEM and MSP is that cMEM requires baseline data, which we selected as the first 100 samples of each register. This might constitute a practical advantage of MSP over cMEM, although we did not analyze the sensitivity of cMEM against different selections of the baseline, as it was out of the scope of this work.

In terms of computational performance, we found that an sLOR-ETA computation takes less than a second, an MSP solution takes approximately 2.5 minutes and the cMEM solutions took an average of 12 minutes each, when using the same standard desktop PC.

### 4.2. Conductivity robustness

sLORETA and MSP algorithms appear to be, in general, robust against scalp and skull conductivity changes for the tested changes of ±15%. Interestingly, for the subjects where some of the sLORETA solutions showed errors when using different conductivity values, the MSP method was robust and vice-versa. This is another indication of the different nature of both methods. In 4 out of the 12 subjects that had good LE, the LE increased when computing the sLORETA solutions with alternative conductivity values, whereas this happened in 6 subjects when using MSP. Another interesting finding was that for one of the three subjects that failed to converge, subject S8, some combinations of the alternative conductivity values tested reduced the LE of the sLORETA solution, even causing the solution to fall inside the VOI. This indicates that the source localization error for this subject might be due to a misspecification of the conductivity values. The other two subjects that initially had large LEs (S4 and S9) showed robust solutions against the other conductivity values evaluated, thus it is likely the LEs in these cases are due to some other reason.

Another interesting observation is the upper-right or bottom-left triangular shape of the reddish regions of the matrices in Fig. 7 in almost all cases where conductivity changes leaded to larger LEs. This is expected, as reducing the conductivity of one tissue and at the same time increasing the other tissue conductivity produces the major difference in the ratio between both tissues, which is known to influence predominantly the electric current distribution. In some cases, the LE matrices have larger LEs in the upper triangle and some other in the lower triangle. These differences indicate that the literature scalp to skull conductivity ratio used is likely lower (in the upper-right LE matrix cases) or larger (in the bottom-left LE matrix cases) with respect to the best fit individual conductivity ratios. However, both methods do not seem to behave similarly in this sense. If one method shows upper or lower triangular LE matrix shape, in most of the cases, the other method shows a flat shape, meaning more robustness against conductivity changes.

Although we obtained good success rate in localizing the spikes using literature conductivity values, the fact that changing 15% of the scalp or skull conductivity values lead to larger LEs (with at least one of the methods) in 8 out of 12 subjects suggests that the use of calibrated conductivity values is preferable. Moreover, the fact that for one subject that originally failed to accurately localize the VOI and a different conductivity specification resulted in success, also supports this idea. This calibration can be done with little additional time burden to the EEG exam using bounded Electrical Impedance Tomography (bElT) (Fernandez-Corazza et al., 2018; Ferree et al., 2000; Goncalves et al., 2003; Rush and Driscoll, 1968). Unfortunately, we did not implement bElT in this study, and that was the main reason for performing this conductivity analysis.

### 4.3. Limitations

One limitation of this study is that we considered the resected region as the ground truth. This region is somewhat large in some subjects and there is the uncertainty of where the true spike generator is located within this volume. As the VOI was marked by the neurosurgeon by hand, this procedure is also subject to human errors, although we estimate the errors due to this fact lower than 5 mm (being pessimistic) as the neurosurgeon that marked the resected volume is the same one that in fact did the resections and he based this marking in all pre- and post-surgery available data. We evaluated performance of the three inverse methods using the alternative VOIs, based on atlas segmentation, to mitigate this limitation. However, this alternative also presents its own limitations (e.g., individual to a normalized space mapping might be subject to errors, and the standard MNI parcellation into Brodmann areas also have its limitations (Geyer et al., 2011)).

The fact that the resected region relieved patients from seizures does not necessarily reflect the fact that the spike originated at the resected region. From a network perspective, seizures are a result of a pathological neural circuit, and disruption of the circuit is just as effective in eliminating seizures. Thus, if the surgery resected a node or a connection of this network, the patient can remain seizure free, even though the spike was generated in a different brain region. However, this is unlikely, given that we generated the forward solution of the source estimates and compared the voltage distribution to the actual EEG potential field and obtaining, in the majority of the cases, good agreement between them. Also, there is the possibility that the spike is generated at the region surrounding a lesion rather than at the lesion itself (Blenkmann et al., 2012). Relatedly, even though we localized interictal spikes, localizing seizure onset is a more accurate indicator of the epileptogenic zone.

Another limitation is the use of standardized electrical conductivities and not subject specific values. It is known that the inverse solvers are sensitive to these parameters (Vanrumste et al., 2000; Wolters et al., 2006). In this study we spanned the scalp and skull conductivity values ±15% and we found that for approximately half of the subjects the solutions seem to be robust against these changes. For other subjects, we found that the solutions were sensitive with respect to using different conductivity values, thus suggesting that, although literature conductivity values produced good results, using bEIT is still recommended. For one of the three subjects that the source localization initially failed, there was an improvement by adjusting the conductivity values, which suggests that the cause of the mis-localization was the use of literature conductivity values. This did not occur for the other two subjects that originally failed to identify the VOI.

The a-priori position of the possible sources of activity is another limitation. In the present research, the gray matter surface was parcellated into patches and one dipole was assigned to each patch. This procedure imposes a restriction to the solution to be on that surface and with normal orientation according to the normal vectors of this surface. In some cases where the source extends to ventral locations not exactly sitting on the cortex, the inverse solution will never be at the true location. The closest cortical location with similar normal orientation will probably get the largest activation, which might lead to localization errors.

A final limitation of this study was the use of “typical” sensor coordinates, based on application of the sensor net with skull fiducial landmarks. Measurement of sensor positions with a 3D digitizer may resolve some of the localization errors in relation to closely adjacent, but functionally distinct, cortical regions.

## 5. Conclusions

Overall, the results suggest that the MSP method is comparable to sLORETA and cMEM methods in performance for this application, at least when using high quality realistic head models. When the MSP method succeeds, the stronger activation peaks are more pronounced than in those found with the sLORETA and cMEM methods. Even when solutions from the three methods are within the VOI, it is not necessarily the case that they exactly agree on the location. This means that these methods are somehow different in nature, as they explain the EEG on the scalp using different assumptions. Thus, we believe that the three methods might be complementary and used together to derive a better description of the possible sources, providing cross-checks to increase confidence in localization when they agree. In practice, MSP will be easier to use than sLORETA (although it will take longer to compute) because the solution is not dependent on hyperparameter selection, and also easier to interpret by a clinician because the solution is much more focal than sLORETA. The facts that the MSP method is five times faster than the cMEM method and that MSP does not require a baseline might constitute practical advantages of MSP.

In terms of the conductivity values of the scalp and the skull, we conclude that assigning literature values is valid when no bEIT data is available, but that subject specific conductivity values is preferred, as half of the studied cases exhibited sensitivity to changes in the conductivity values.

Finally, we described a simple algorithm (based on a comparison between the actual EEG and the synthetic EEG explained, as done empirically by some experts) that can be implemented to determine the best hyperparameter (for sLORETA) and most relevant source polarity (for any method).

## Supplementary Material

2

3

4

5

6

7

8

9

10

11

12

13

Subject_by_ Subject details analysis - Supp.materials

Appendices A and B Final

## Figures and Tables

**Fig. 3.**
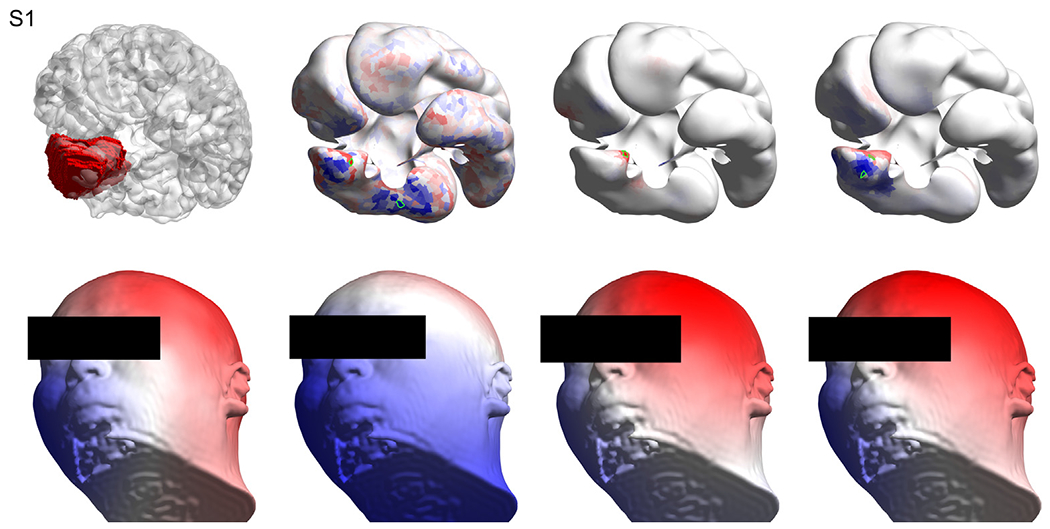
Results for subject S1. Top left: the volume of interest (in red) as marked by the neurosurgeon on the T1 image, overlaid with the wrinkled cortex. Top row, second, third and fourth columns show the solutions obtained with sLORETA, MSP and cMEM respectively, where the selected patches based on the best artificial versus real EEG match are highlighted in green. Bottom left: the interpolated (Oostendorp et al., 1989) real EEG on the scalp at the time point of interest. Bottom row, second, third and fourth columns show the artificial EEG modeled by placing dipoles centered at the patches marked in green in the top row for sLORETA, MSP and cMEM, respectively. Methods: standardized low-resolution brain electromagnetic tomography (sLORETA), multiple sparse priors (MSP) and coherent maximum entropy on the mean (cMEM). (For interpretation of the references to colour in this figure legend, the reader is referred to the web version of this article.)

**Fig. 4.**
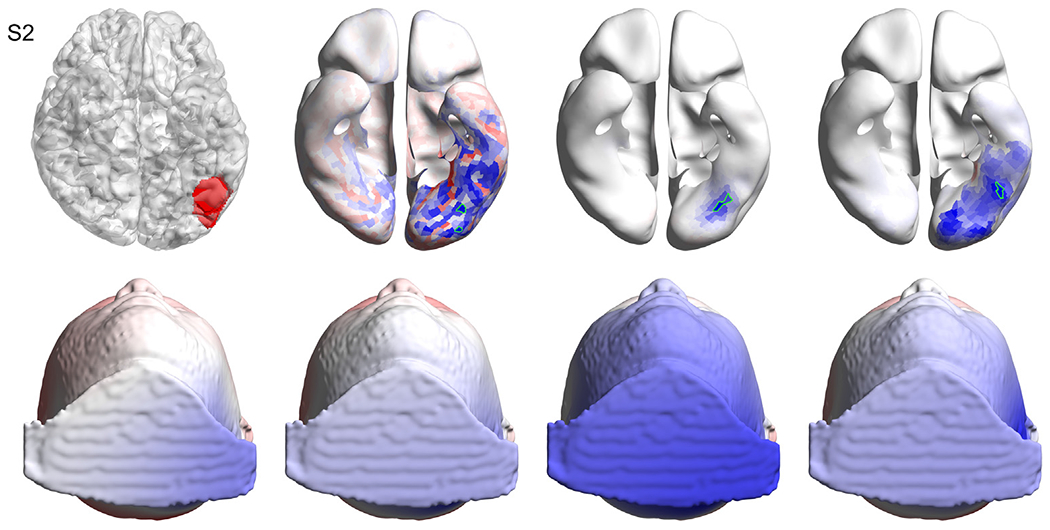
Results for subject S2. Top left: the volume of interest (in red) as marked by the neurosurgeon on the T1 image, overlaid with the wrinkled cortex. Top row, second, third and fourth columns show the solutions obtained with sLORETA, MSP and cMEM respectively, where the selected patches based on the best artificial versus real EEG match are highlighted in green. Bottom left: the interpolated real EEG on the scalp at the time point of interest. Bottom row, second, third and fourth columns show the artificial EEG modeled by placing dipoles centered at the patches marked in green in the top row for sLORETA, MSP and cMEM, respectively. Methods: standardized low-resolution brain electromagnetic tomography (sLORETA), multiple sparse priors (MSP) and coherent maximum entropy on the mean (cMEM). (For interpretation of the references to colour in this figure legend, the reader is referred to the web version of this article.)

**Fig. 5.**
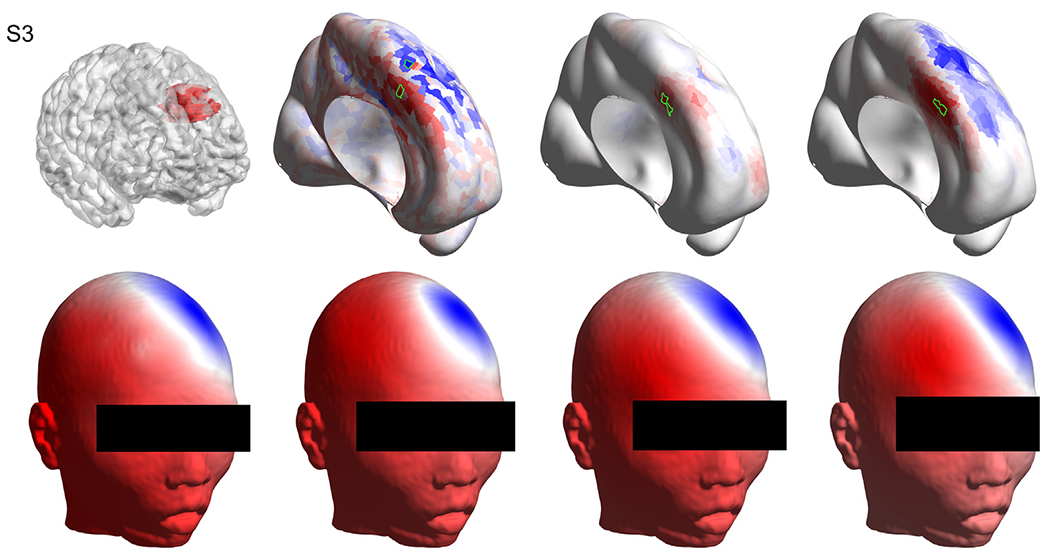
Results for subject S3. Top left: the volume of interest (in red) as marked by the neurosurgeon on the T1 image, overlaid with the wrinkled cortex. Top row, second, third and fourth columns show the solutions obtained with sLORETA, MSP and cMEM respectively, where the selected patches based on the best artificial versus real electroencephalography (EEG) match are highlighted in green. Bottom left: the interpolated real EEG on the scalp at the time point of interest. Bottom row, second, third and fourth columns show the artificial EEG modeled by placing dipoles centered at the patches marked in green in the top row for sLORETA, MSP and cMEM, respectively. Methods: standardized low-resolution brain electromagnetic tomography (sLORETA), multiple sparse priors (MSP) and coherent maximum entropy on the mean (cMEM). (For interpretation of the references to colour in this figure legend, the reader is referred to the web version of this article.)

**Table 1:** Patient information. The used “time point” is relative to the spike peak.

Subject	Gender	Age [years]	Volume of Interest (VOI) Location	VOI volume [cm^3^]	Time point [ms]	Number of bad Chanels
S1	M	26	R. hippocampus	51	−12	4
S2	M	21	L. temporal lobe	7	−12	0
S3	M	17	L. medial frontal lobe	14	−12	0
S4	M	7	R. central sulcus	1	−12	12
S5	M	37	L. temporal anterior lobe	38	−16	0
S6	M	19	R. ventro-medial prefrontal lobe	20	−12	2
S7	M	28	L. medial-frontal lobe	42	−10	7
S8	F	15	L. anterior temporal lobe	28	−24	0
S9	M	26	R. posterior temporal lobe	34	−17	0
S10	M	19	R. medial temporal lobe	58	−12	3
S11	F	45	R. medial temporal lobe	42	−6	0
S12	F	28	L. medial temporal and left frontal lobes	36	−16	0
S13	M	25	R. frontal lobe	90	−5	0
S14	M	32	L. parahippo-campal gyrus	17	−12	0
S15	F	36	L. anterior temporal lobe	34	−12	0

**Table 2:** Localization error (LE) for all subjects and for the three studied methods: standardized low-resolution brain electromagnetic tomography (sLORETA), multiple sparse priors (MSP) and coherent maximum entropy on the mean (cMEM). The best polarities and hyperparameters (the latter only for sLORETA) are also shown. The volume of interest (VOI) location of Table 1 is presented as a reference.

Subj.	VOI Location	sLORETA			MSP		cMEM	
		LE* [mm]	Polarity	Best Hyp.	LE* [mm]	Polarity	LE* [mm]	Polarity
S1	R. hippocampus	**<1**	Both	0.001	**<1**	Max	**<1**	Both
S2	L. temporal lobe	**4**	Min	10	**<1**	Min	**3**	Min
S3	L. medial frontal lobe	**<1**	Both	1	**<1**	Max	**5**	Max
S4	R. central sulcus	22	Min	10	46	Min	21	Both
S5	L. temp. ant. Lobe	**<1**	Both	10	**<1**	Both	**<1**	Max
S6	R. ventro-medial prefront. Lobe	**<1**	Min	1	**<1**	Min	5	Both
S7	L. medial-frontal lobe	**<1**	Both	1	**<1**	Min	**<1**	Min
S8	L. anterior temporal lobe	44	Max	10	***13***	Max	40	Min
S9	R. posterior temporal lobe	20	Min	10	60	Min	***15***	Min
S10	R. medial temporal lobe	**<1**	Max	0.1	**<1**	Min	**<1**	Min
S11	R. medial temporal lobe	**3**	Both	1	**<1**	Max	**<1**	Min
S12	L. medial temporal and left frontal lobes	**<1**	Min	0.1	**<1**	Min	22	Min
S13	R. frontal lobe	**<1**	Max	0.01	**<1**	Min	**<1**	Both
S14	L. parahippo-campal gyrus	**<1**	Both	0.01	**3**	Min	23	Min
S15	L. anterior temporal lobe	**<1**	Both	1	**<1**	Max	**<1**	Both

* Bold text indicates low LE (less than 5 mm), italic indicates intermediate LE (between 5 and 15 mm), and regular font style indicates large LE (more than 15 mm).

**Table 3:** Brodmann areas (BAs) of the volumes of interest (VOIs) and of the centers of mass (CoMs) for the subjects that had at least one algorithm failing in Table 2. The third column shows the BAs of the VOI, and the fourth, fifth and sixth columns show the BAs of the CoMs obtained with each inverse method: standardized low-resolution brain electromagnetic tomography (sLORETA), multiple sparse priors (MSP) and coherent maximum entropy on the mean (cMEM). Numbers 1–47 correspond to the BAs of left hemisphere, 48 is the left caudate, 49: left putamen, 50: left thalamus, 51: left globus pallidus, 52: left nucleus accumbens, 53: left amygdala, and 54–55: left hippocampus. The same numbers plus 100 are the analogous brain regions on the right hemisphere.

Subject	VOI location (native space)	BAs involved in VOI	CoM sLORETA BA*	CoM MSP BA*	CoM cMEM BA*
S4	R. central sulcus	101, 104, 106	113	136	**104**
S8	L. anterior temporal lobe	11, 20, 21, 34, 36, 38, 47, 53, 54	24	**11**	45
S9	R. posterior temporal lobe	118, 119, 121, 122, 137, 138, 139, 140, 141	136	37	**139**
S12	L. medial temporal and left frontal lobes	13, 20, 21, 22, 34, 36, 37, 38, 47, 48, 49, 53, 54	**53**	**49**	11
S14	L. parahippo-campal gyrus	11, 13, 20, 34, 36, 38, 47, 49, 53, 54	**34**	**49**	**11**

* Bold text indicates that the BA of the CoM is part of the VOI and regular font style indicates that it is not.

## HIGHLIGHTS

- Bayesian multiple sparse priors (MSP) inverse method accurately localizes epilepsy foci.
- MSP solutions are more focal and easier to use than sLORETA inverse method.
- MSP inverse method showed similar or better accuracy than coherent maximum entropy on the mean inverse method and is faster to compute.

## Footnotes

2 $NRDM\left(v,w\right)=\sqrt{\Sigma_{i}{\left(\frac{v_{i}}{{\left|v\right|}_{2}}-\frac{w_{i}}{{\left|w\right|}_{2}}\right)}^{2}}$, where **v** and **w** are two arbitrary vectors.

## References

[R1] Baumann SB, Wozny DR, Kelly SK, Meno FM. The electrical conductivity of human cerebrospinal fluid at body temperature. IEEE Trans Biomed Eng 1997;44:220–3. 10.1109/10.554770. 9216137

[R2] Beltrachini L, von Ellenrieder N, Muravchik CH. General bounds for electrode mislocation on the EEG inverse problem. Comput Methods Programs Biomed 2011;103:1–9. 10.1016/j.cmpb.2010.05.008. 20599288

[R3] Blenkmann A, Seifer G, Princich JP, Consalvo D, Kochen S, Muravchik C. Association between equivalent current dipole source localization and focal cortical dysplasia in epilepsy patients. Epilepsy Res 2012;98:223–31. 10.1016/j.eplepsyres.2011.09.018. 22018907

[R4] Blume WT, Lüders HO, Mizrahi E, Tassinari C, Van Emde Boas W, Engel J. Glossary of descriptive terminology for ictal semiology: report of the ILAE task force on classification and terminology. Epilepsia 2002;42:1212–8. 10.1046/j.1528-1157.2001.22001.x. 11580774

[R5] Brodbeck V, Spinelli L, Lascano AM, Wissmeier M, Vargas M-I, Vulliemoz S, Electroencephalographic source imaging: a prospective study of 152 operated epileptic patients. Brain 2011;134:2887–97. 10.1093/brain/awr243. 21975586 PMC3187544

[R6] Chowdhury RA, Merlet I, Birot G, Kobayashi E, Nica A, Biraben A, Wendling F, Lina JM, Albera L, Grova C. Complex patterns of spatially extended generators of epileptic activity: comparison of source localization methods cMEM and 4-ExSo-MUSIC on high resolution EEG and MEG data. Neuroimage 2016;143:175–95. 10.1016/j.neuroimage.2016.08.044. 27561712

[R7] Cosandier-Rimélé D, Ramantani G, Zentner J, Schulze-Bonhage A, Dümpelmann M. A realistic multimodal modeling approach for the evaluation of distributed source analysis: application to sLOReTa. J Neural Eng 2017;14:056008. 10.1088/1741-2552/aa7db1. 28677591

[R8] Daunizeau J, Vaudano AE, Lemieux L. Bayesian multi-modal model comparison: a case study on the generators of the spike and the wave in generalized spike-wave complexes. Neuroimage 2010;49:656–67. 10.1016/j.neuroimage.2009.06.048. 19559798

[R9] Meijs JWH, Weier OW, Peters MJ, Van Oosterom A. On the numerical accuracy of the boundary element method (EEG application). IEEE Trans. Biomed. Eng 1989;36 (10):1038–49. 10.1109/10.40805. 2793196

[R10] de Munck JC, Faes TJ, Heethaar RM. The boundary element method in the forward and inverse problem of electrical impedance tomography. IEEE Trans Biomed Eng 2000;47:792–800. 10.1109/10.844230. 10833854

[R11] Engel J A proposed diagnostic scheme for people with epileptic seizures and with epilepsy: report of the ILAE task force on classification and terminology. Epilepsia 2001;42:796–803. 10.1046/j.1528-1157.2001.10401.x. 11422340

[R12] Feng R, Hu J, Wu J, Lang L, Ma C, Sun B, Gu X, Pan L. Accurate source imaging based on high resolution scalp electroencephalography and individualized finite difference head models in epilepsy pre-surgical workup. Seizure 2018;59:126–31. 10.1016/j.seizure.2018.05.009. 29843085

[R13] Fernandez-Corazza M, Turovets S, Luu P, Price N, Muravchik CH, Tucker D, Skull modeling effects in conductivity estimates using parametric electrical impedance tomography. IEEE Trans Biomed Eng 2018;65:1785–97. 10.1109/TBME.2017.2777143. 29989921

[R14] Ferree TC, Eriksen KJ, Tucker DM. Regional head tissue conductivity estimation for improved EEG analysis. IEEE Trans Biomed Eng 2000;47:1584–92. 10.1109/10.887939. 11125593

[R15] Fiederer LDJ, Vorwerk J, Lucka F, Dannhauer M, Yang S, Dümpelmann M, Schulze-Bonhage A, Aertsen A, Speck O, Wolters CH, Ball T. The role of blood vessels in high-resolution volume conductor head modeling of EEG. Neuroimage 2016;128:193–208. 10.1016/j.neuroimage.2015.12.041. 26747748 PMC5225375

[R16] Friston K, Harrison L, Daunizeau J, Kiebel S, Phillips C, Trujillo-Barreto N, Henson R, Flandin G, Mattout J. Multiple sparse priors for the M/EEG inverse problem. Neuroimage 2008;39:1104–20. 10.1016/j.neuroimage.2007.09.048. 17997111

[R17] Gabriel S, Lau RW, Gabriel C. The dielectric properties of biological tissues: II. Measurements in the frequency range 10 Hz to 20 GHz. Phys Med Biol 1996;41:2251–69. 8938025 10.1088/0031-9155/41/11/002

[R18] Geyer S, Weiss M, Reimann K, Lohmann G, Turner R. Microstructural parcellation of the human cerebral cortex – from Brodmann’s post-mortem map to in vivo mapping with high-field magnetic resonance imaging. Front Hum Neurosci 2011;5. 10.3389/fnhum.2011.00019. 21373360 PMC3044325

[R19] Goncalves SI, de Munck JC, Verbunt JPA, Bijma F, Heethaar RM, Lopes da Silva F. In vivo measurement of the brain and skull resistivities using an EIT-based method and realistic models for the head. IEEE Trans Biomed Eng 2003;50:754–67. 10.1109/TBME.2003.812164. 12814242

[R20] Grech R, Cassar T, Muscat J, Camilleri KP, Fabri SG, Zervakis M, Xanthopoulos P, Sakkalis V, Vanrumste B. Review on solving the inverse problem in EEG source analysis. J NeuroEng Rehabil 2008;5:25. 10.1186/1743-0003-5-25. 18990257 PMC2605581

[R21] Grova C, Daunizeau J, Lina J-M, Bénar CG, Benali H, Gotman J. Evaluation of EEG localization methods using realistic simulations of interictal spikes. Neuroimage 2006;29:734–53. 10.1016/j.neuroimage.2005.08.053. 16271483

[R22] Hallez H, Vanrumste B, Grech R, Muscat J, De Clercq W, Vergult A, D’Asseler Y, Camilleri KP, Fabri SG, Van Huffel S, Lemahieu I. Review on solving the forward problem in EEG source analysis. J NeuroEng Rehabil 2007;4:46. 10.1186/1743-0003-4-46. 18053144 PMC2234413

[R23] Hämäläinen MS, Ilmoniemi RJ. Interpreting magnetic fields of the brain: minimum norm estimates. Med Biol Eng Comput 1994;32:35–42. 10.1007/BF02512476. 8182960

[R24] Heers M, Chowdhury RA, Hedrich T, Dubeau F, Hall JA, Lina J-M, Grova C, Kobayashi E. Localization accuracy of distributed inverse solutions for electric and magnetic source imaging of interictal epileptic discharges in patients with focal epilepsy. Brain Topogr 2016;29:162–81. 10.1007/s10548-014-0423-1. 25609211

[R25] Henson RN, Flandin G, Friston KJ, Mattout J. A Parametric Empirical Bayesian framework for fMRI-constrained MEG/EEG source reconstruction. Hum Brain Mapp 2010;31:1512–31. 10.1002/hbm.20956. 20091791 PMC2941720

[R26] Jehi L The epileptogenic zone: concept and definition. Epilepsy Curr 2018;18:12–6. 10.5698/1535-7597.18.1.12. 29844752 PMC5963498

[R27] Kiebel SJ, Friston KJ. Statistical parametric mapping for event-related potentials (II): a hierarchical temporal model. Neuroimage 2004;22:503–20. 10.1016/j.neuroimage.2004.02.013 15193579

[R28] Li K, Papademetris X, Tucker DM. BrainK for structural image processing: creating electrical models of the human head. Comput Intell Neurosci 2016;2016:1–25. 10.1155/2016/1349851. PMC4884832 27293419

[R29] Lindenblatt G, Silny J. A model of the electrical volume conductor in the region of the eye in the ELF range. Phys Med Biol 2001;46:3051–9. 11720363 10.1088/0031-9155/46/11/319

[R30] Lüders HO, Najm I, Nair D, Widdess-Walsh P, Bingman W. The epileptogenic zone: general principles. Epileptic Disord 2006;8(Suppl 2):S1–9. 17012067

[R31] Malmivuo J, Plonsey R. Bioelectromagnetism: principles and applications of bioelectric and biomagnetic fields. Oxford University Press; 1995.

[R32] Martinez-Vargas JD, Strobbe G, Vonck K, van Mierlo P, Castellanos-Dominguez G. Improved localization of seizure onset zones using spatiotemporal constraints and time-varying source connectivity. Front Neurosci 2017;11. 10.3389/fnins.2017.00156. 28428738 PMC5382162

[R33] Oostendorp TF, van Oosterom A, Huiskamp G. Interpolation on a triangulated 3D surface. J Comput Phys 1989;80:331–43. 10.1016/0021-9991(89)90103-4.

[R34] Oostendorp TFF, Delbeke J, Stegeman DFF. The conductivity of the human skull: results of in vivo and in vitro measurements. IEEE Trans Biomed Eng 2000;47:1487–92. 10.1109/TBME.2000.880100. 11077742

[R35] Pascual-Marqui RD. Standardized low-resolution brain electromagnetic tomography (sLORETA): technical details. Methods Find. Exp. Clin. Pharmacol. 2002;24(Suppl D):5–12. 12575463

[R36] Pascual-Marqui RD, Michel CM, Lehmann D. Low resolution electromagnetic tomography: a new method for localizing electrical activity in the brain. Int J Psychophysiol 1994;18:49–65. 10.1016/0167-8760(84)90014-X. 7876038

[R37] Plummer C, Wagner M, Fuchs M, Vogrin S, Litewka L, Farish S, Bailey C, Harvey AS, Cook MJ. Clinical utility of distributed source modelling of interictal scalp EEG in focal epilepsy. Clin Neurophysiol 2010;121:1726–39. 10.1016/j.clinph.2010.04.002 20457537

[R38] Ramon C, Schimpf PH, Haueisen J. Influence of head models on EEG simulations and inverse source localizations. Biomed Eng Online 2006;5:10. 10.1186/1475-925X-5-10. 16466570 PMC1389789

[R39] Rush S, Driscoll DA. Current distribution in the brain from surface electrodes. Anesth Analg 1968;47:717–23. 4972743

[R40] Sander JW. The epidemiology of epilepsy revisited. Curr Opin Neurol 2003;16:165–70. 10.1097/01.wco.0000063766.15877.8e. 12644744

[R41] Sekihara K, Nagarajan SS. Adaptive spatial filters for electromagnetic brain imaging. Berlin: Springer; 2008.

[R42] Spencer SS. Neural networks in human epilepsy: evidence of and implications for treatment. Epilepsia 2002;43:219–27. 10.1046/j.1528-1157.2002.26901.x. 11906505

[R43] Tadel F, Baillet S, Mosher JC, Pantazis D, Leahy RM. Brainstorm: a user-friendly application for MEG/EEG analysis. Comput Intell Neurosci 2011;2011:1–13. 10.1155/2011/879716. 21584256 PMC3090754

[R44] Turovets S, Volkov V, Zherdetsky A, Prakonina A, Malony AD. A 3D finite-difference BiCG iterative solver with the fourier-jacobi preconditioner for the anisotropic EIT/EEG forward problem. Comput Math Methods Med 2014;2014:1–12. 10.1155/2014/426902. PMC3913502 24527060

[R45] Vanrumste B, Van Hoey G, de Walle R, D’Havé M, Lemahieu I, Boon P, Van de Walle R, D’Havé M, Lemahieu I, Boon P, de Walle R, D’Havé M, Lemahieu I, Boon P, Van de Walle R, D’Havé M, Lemahieu I, Boon P, de Walle R, D’Havé M, Lemahieu I, Boon P. Dipole location errors in electroencephalogram source analysis due to volume conductor model errors. Med Biol Eng Comput 2000;38:528–34. 10.1007/BF02345748. 11094809

[R46] Wagner M, Fuchs M, Kastner J. Evaluation of sLORETA in the presence of noise and multiple sources. Brain Topogr 2003;16:277–80. 10.1023/B:BRAT.0000032865.58382.62. 15379227

[R47] Wolters CH, Anwander A, Tricoche X, Weinstein D, Koch MA, MacLeod RS. Influence of tissue conductivity anisotropy on EEG/MEG field and return current computation in a realistic head model: a simulation and visualization study using high-resolution finite element modeling. Neuroimage 2006;30:813–26. 10.1016/j.neuroimage.2005.10.014. 16364662

