# Supplementary figures and images for: Source localization of epileptic spikes using Multiple Sparse Priors
Source: Clin Neurophysiol. Author manuscript; Available in PMC 2021 Mar 18. (PMC7971150; doi:10.1016/j.clinph.2020.10.030)

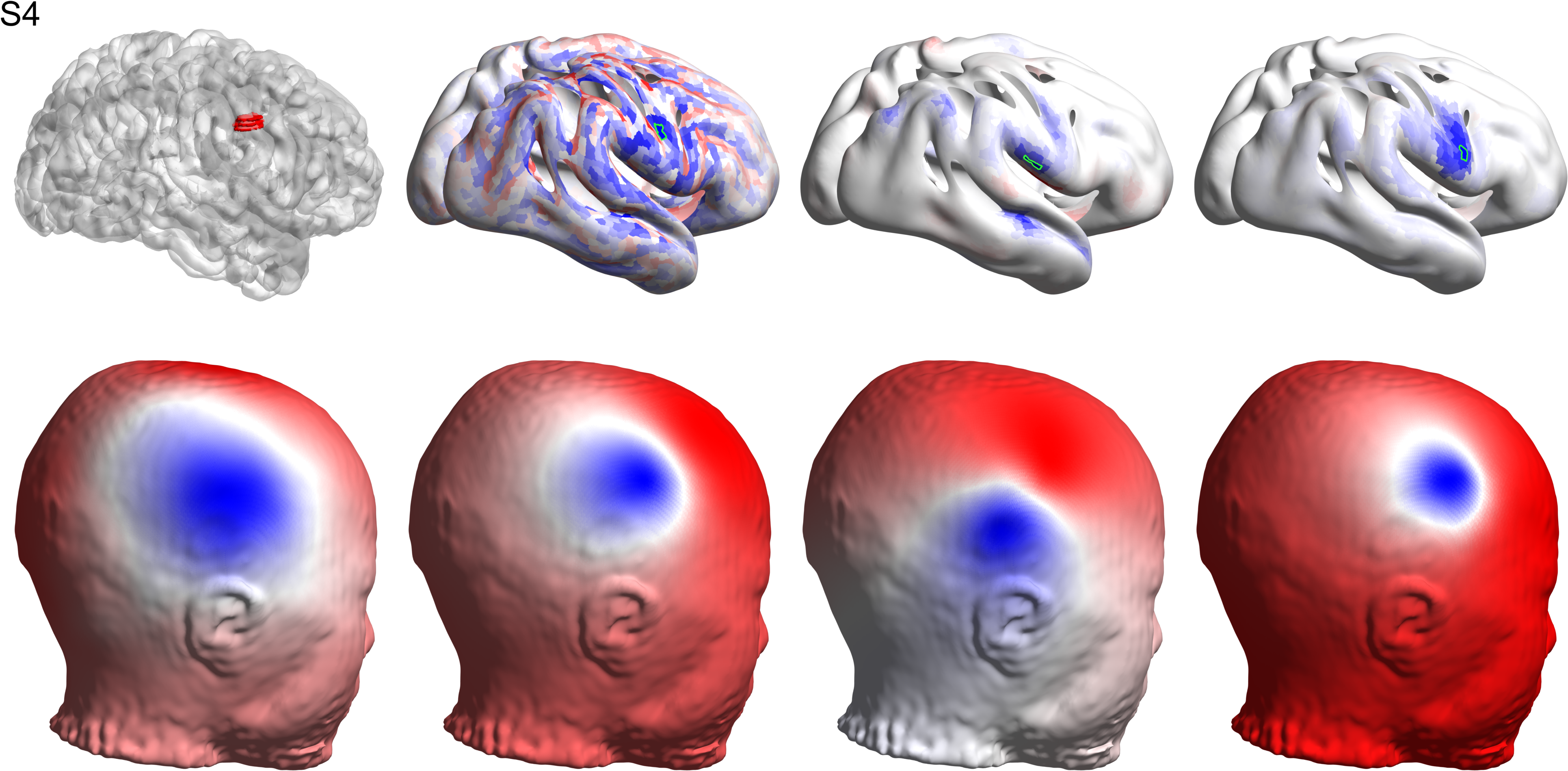

Supplement: 2 [file NIHMS1666397-supplement-2.png]

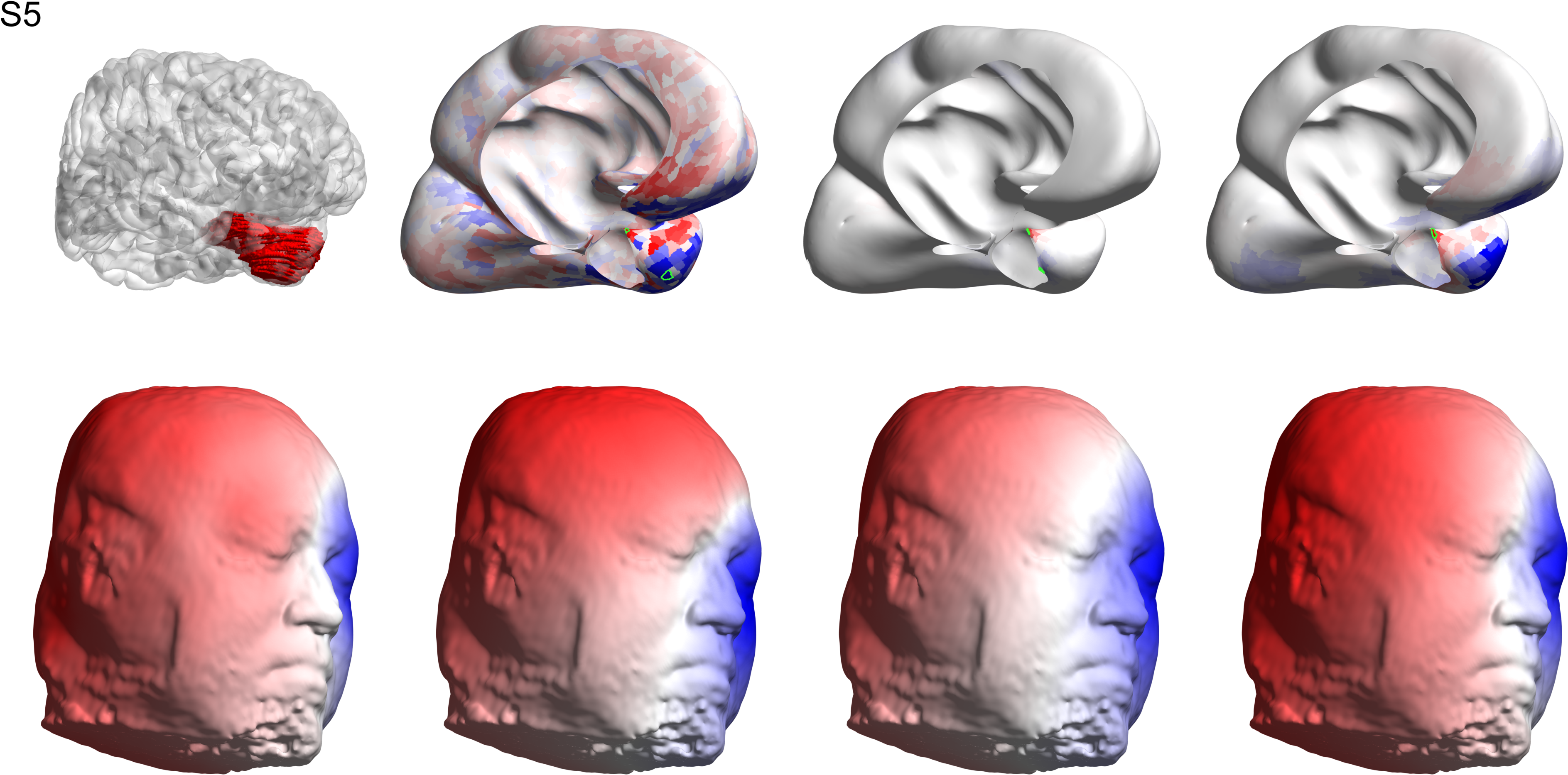

Supplement: 3 [file NIHMS1666397-supplement-3.png]

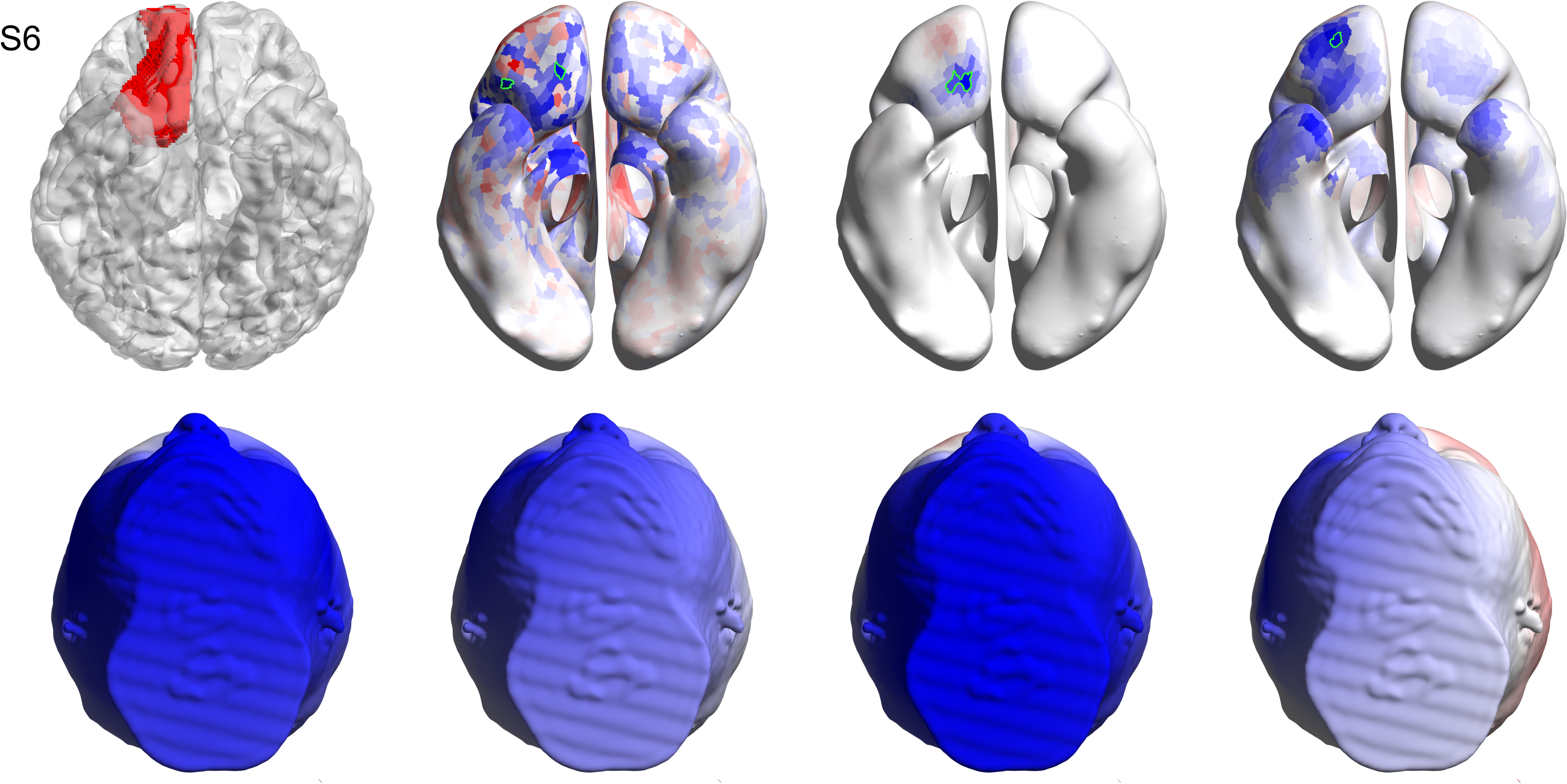

Supplement: 4 [file NIHMS1666397-supplement-4.png]

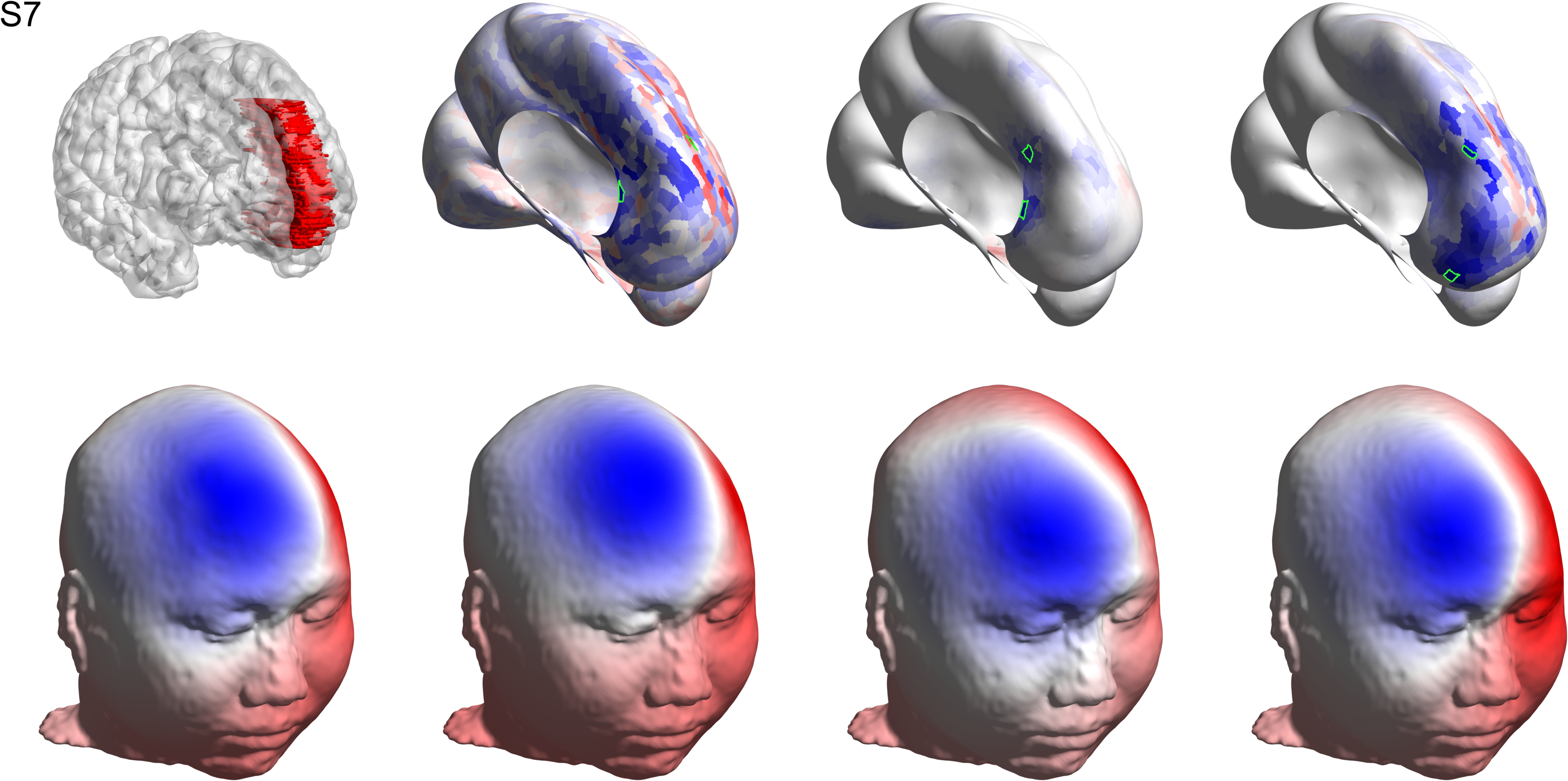

Supplement: 5 [file NIHMS1666397-supplement-5.png]

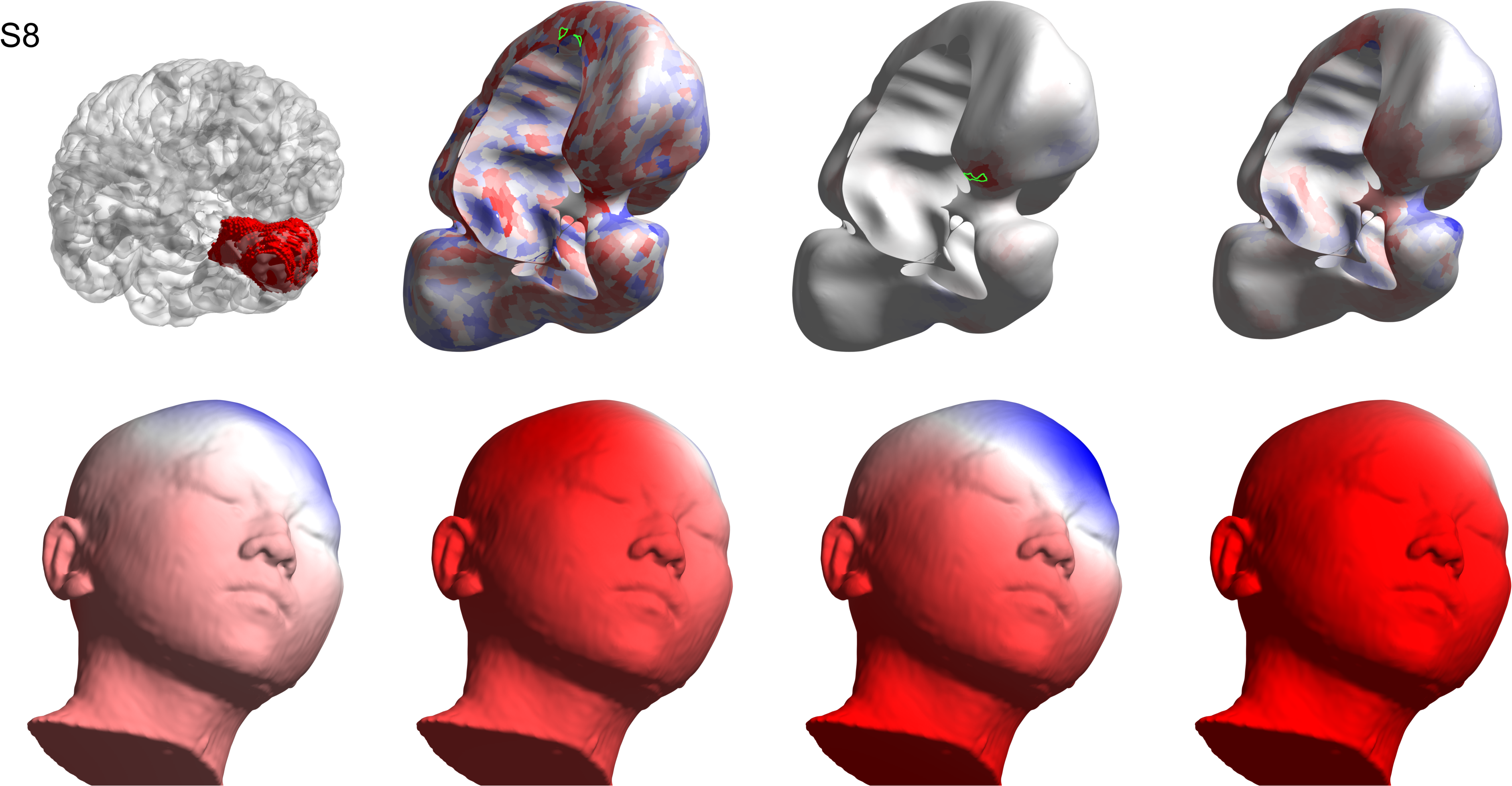

Supplement: 6 [file NIHMS1666397-supplement-6.png]

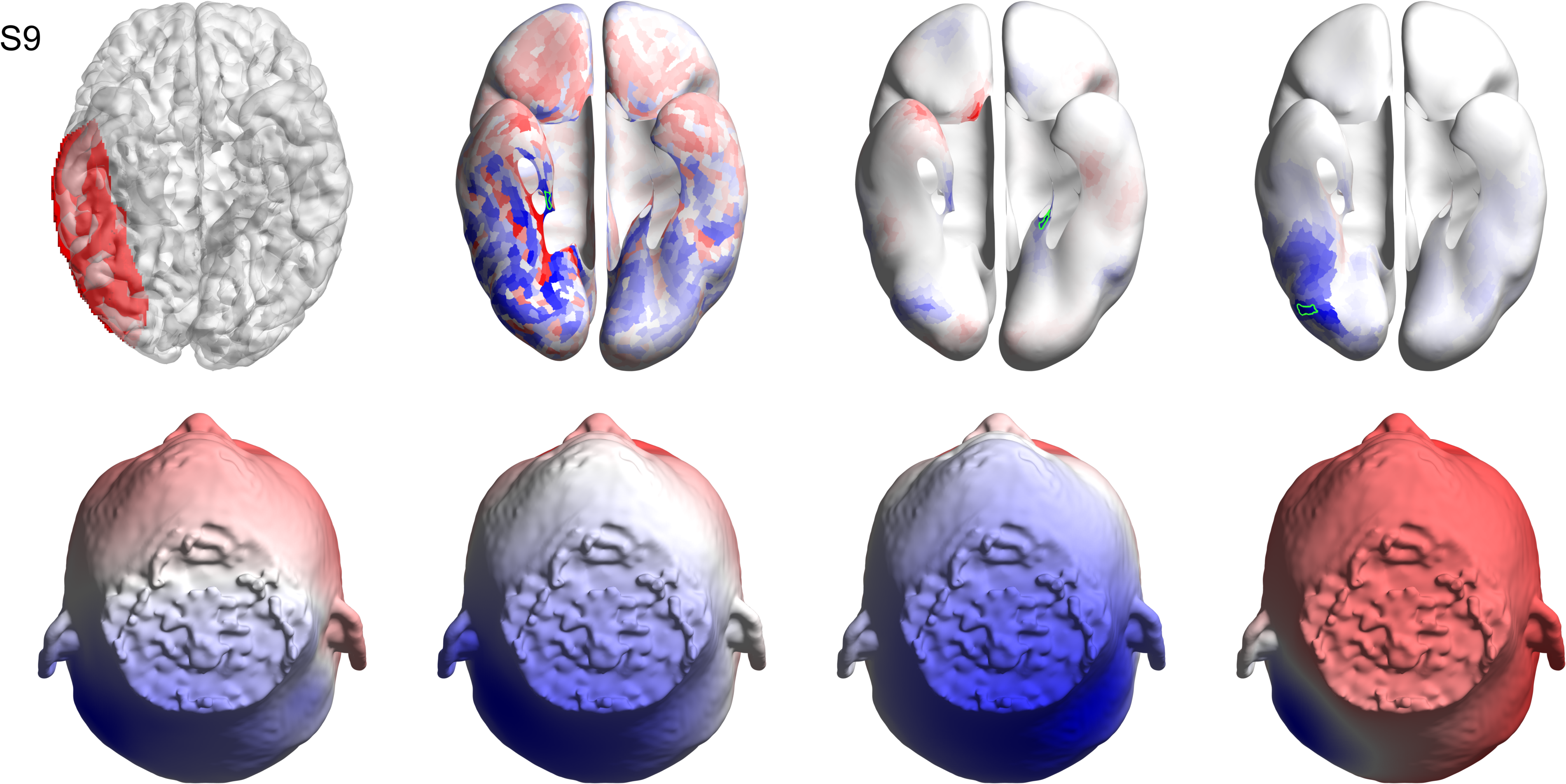

Supplement: 7 [file NIHMS1666397-supplement-7.png]

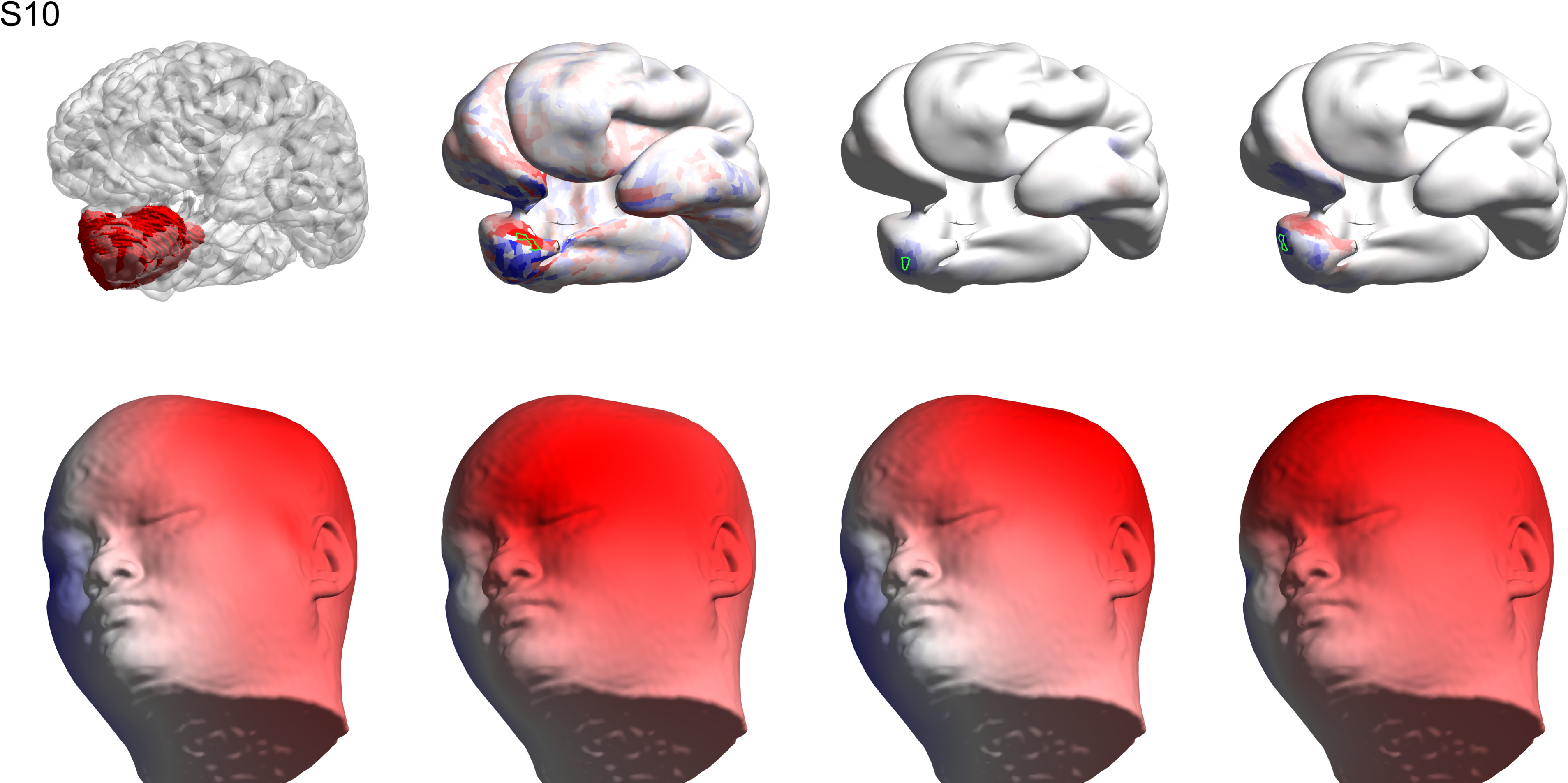

Supplement: 8 [file NIHMS1666397-supplement-8.png]

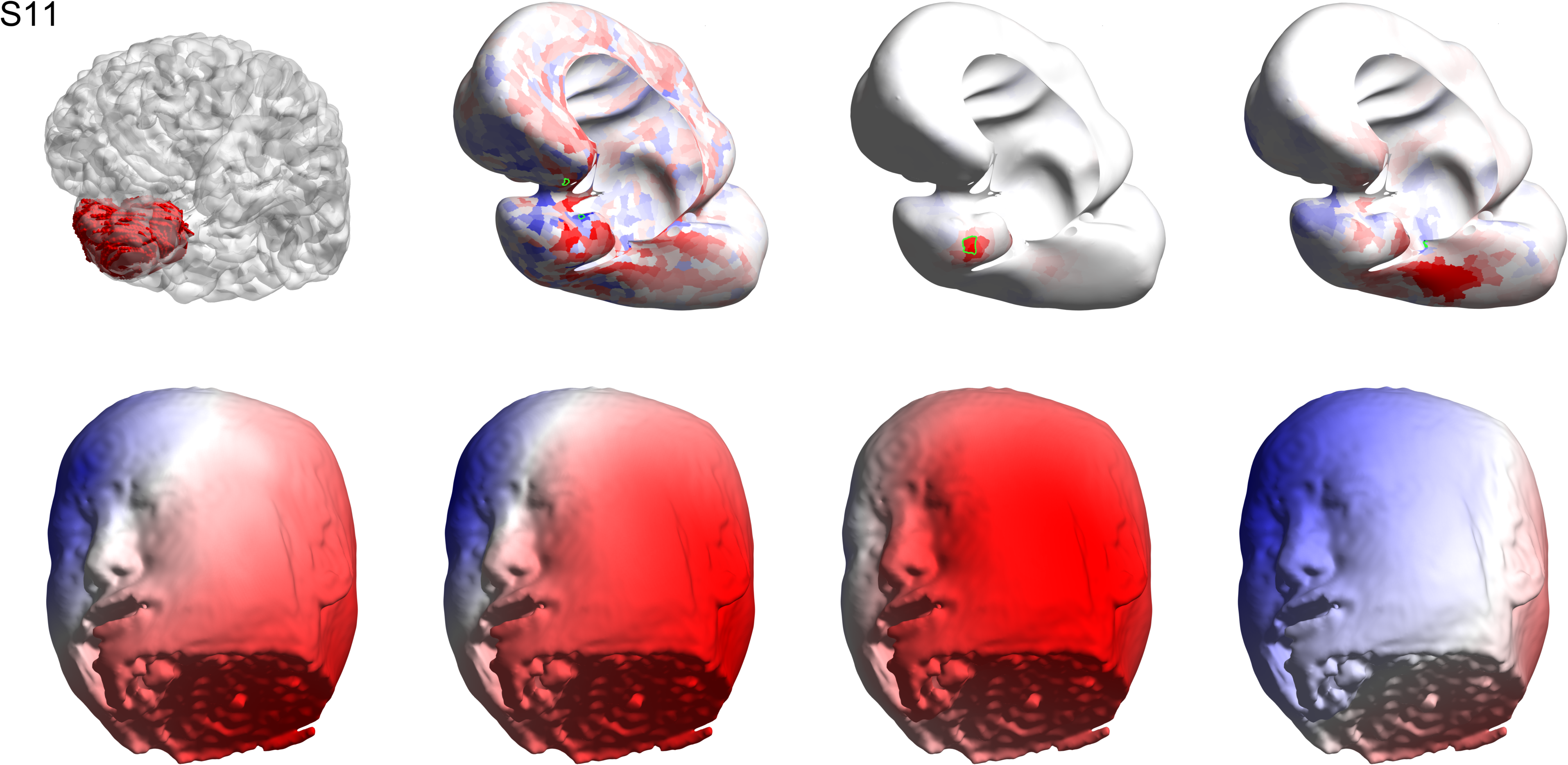

Supplement: 9 [file NIHMS1666397-supplement-9.png]

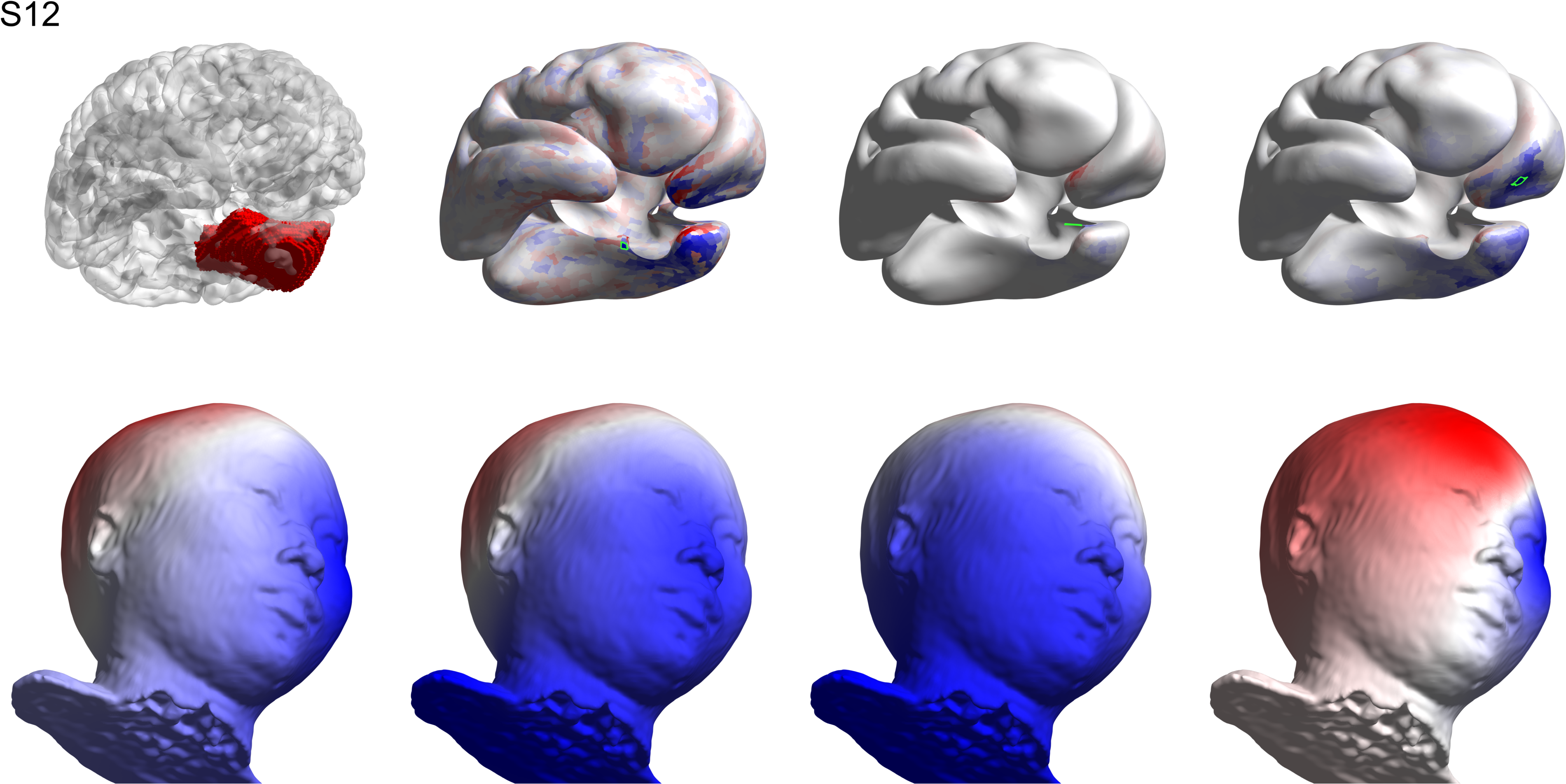

Supplement: 10 [file NIHMS1666397-supplement-10.png]

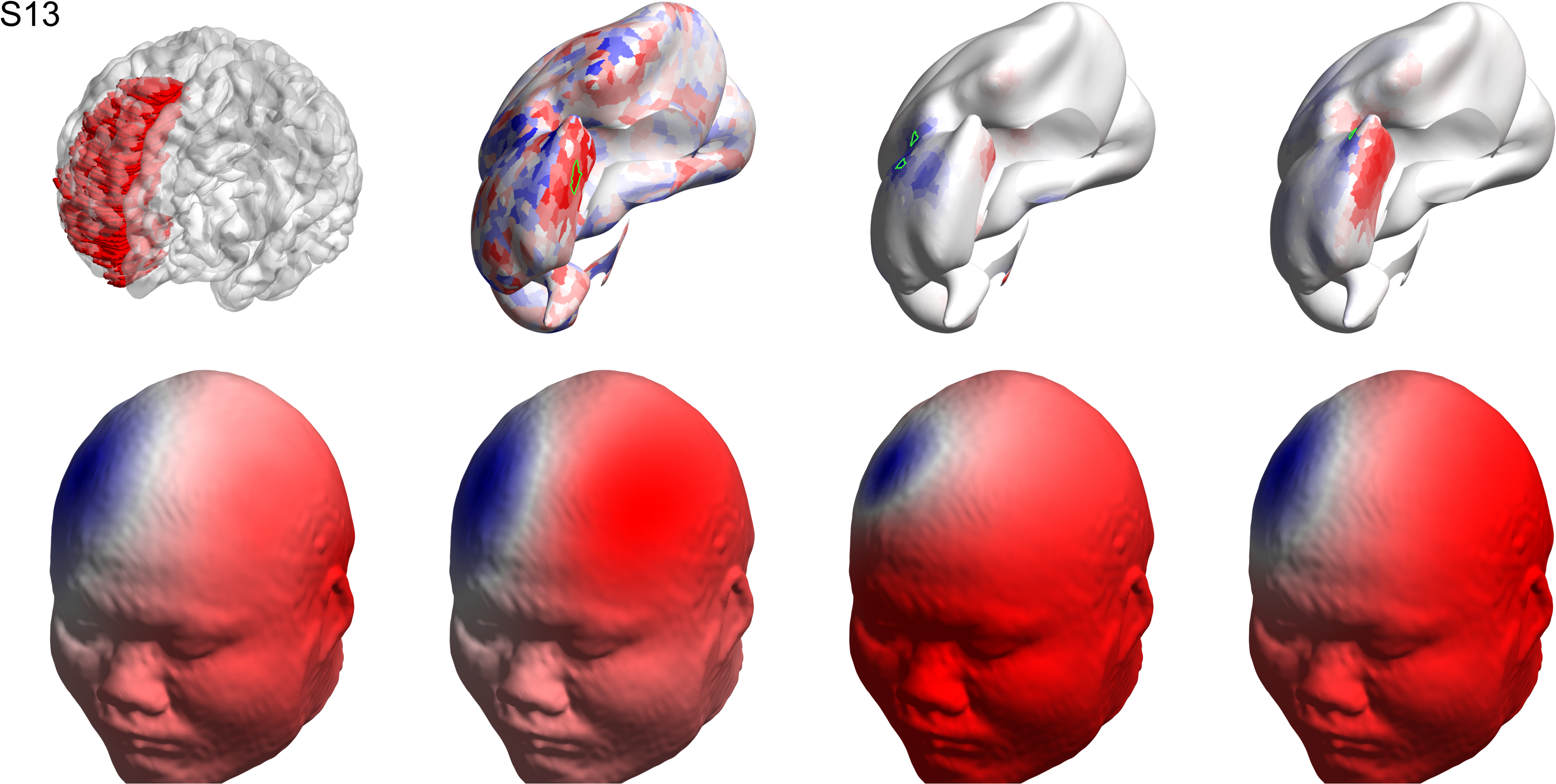

Supplement: 11 [file NIHMS1666397-supplement-11.png]

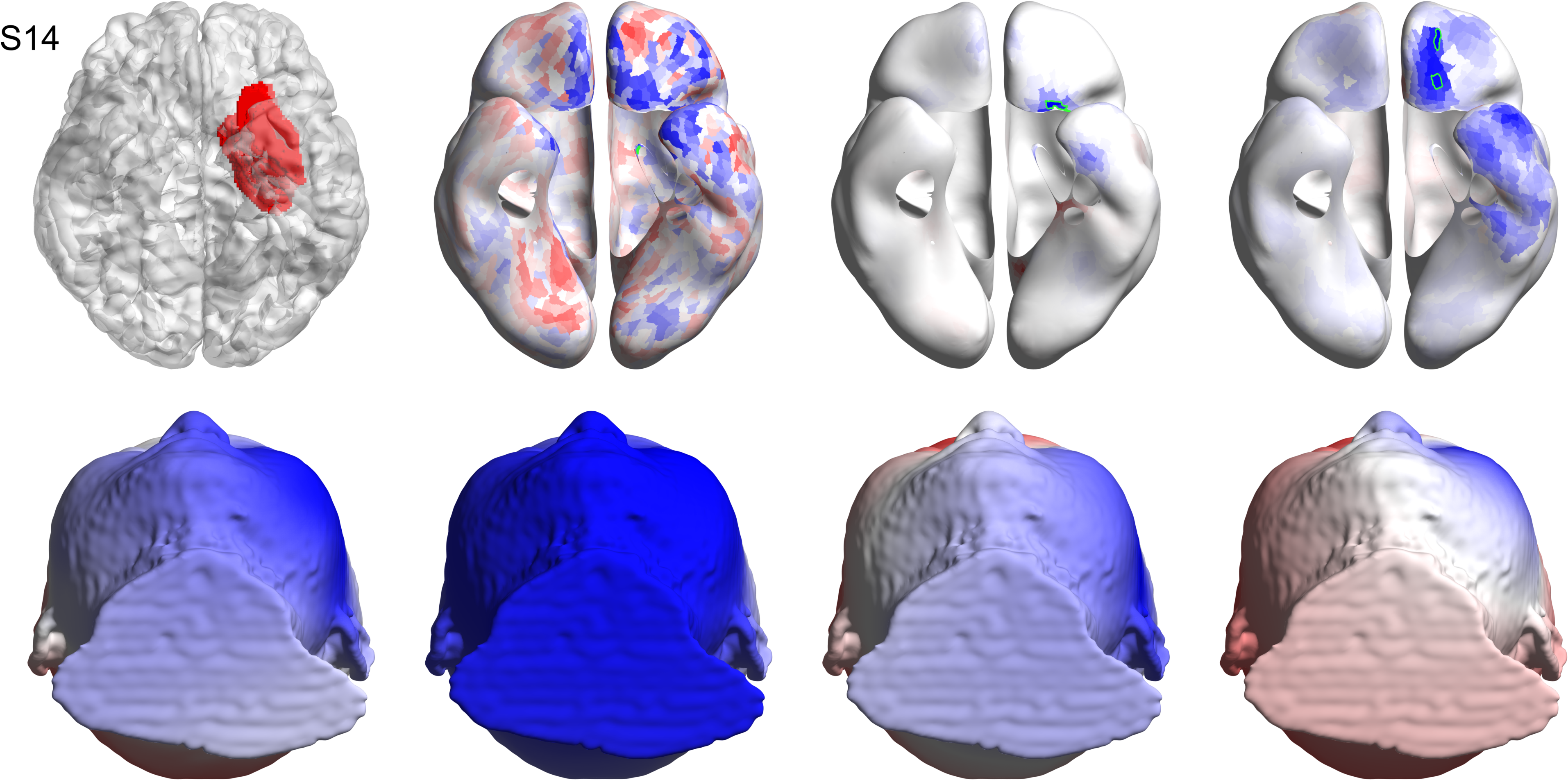

Supplement: 12 [file NIHMS1666397-supplement-12.png]

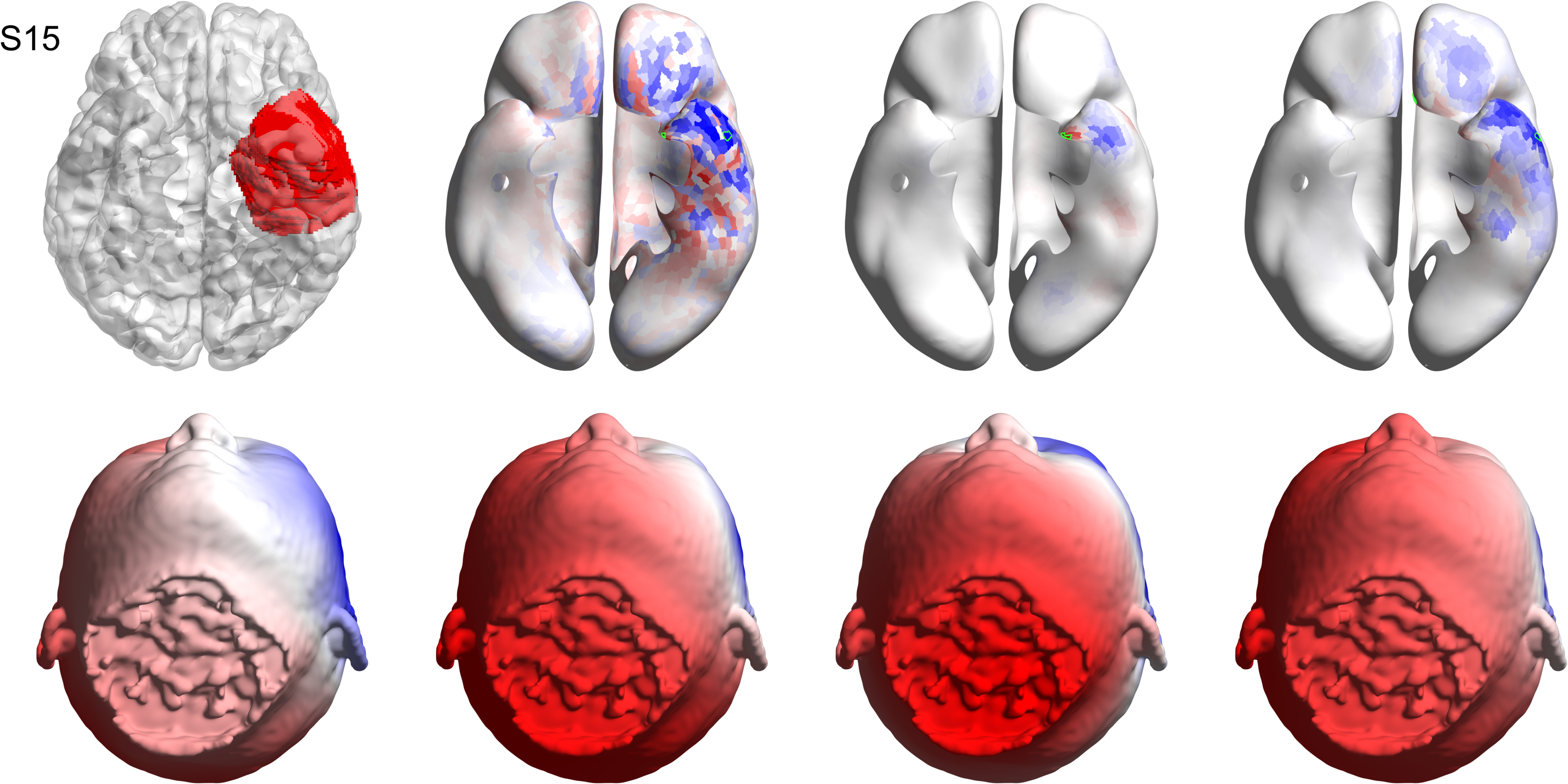

Supplement: 13 [file NIHMS1666397-supplement-13.png]
