# Supplementary material for: Source localization of epileptic spikes using Multiple Sparse Priors
Source: Clin Neurophysiol. Author manuscript; Available in PMC 2021 Mar 18. (PMC7971150; doi:10.1016/j.clinph.2020.10.030)
Supplement: Subject_by_ Subject details analysis - Supp.materials [file NIHMS1666397-supplement-Subject_by__Subject_details_analysis_-_Supp_materials.pdf]

1 Supplementary Material

Here we briefly analyze each subject based on the results presented in Fig. 3 for subject S1, Fig. 4 for S2, Fig. 5 for S3, supplementary Fig. SF1 for S4, supplementary Fig. SF2 for S5, supplementary Fig. SF3 for S6, supplementary Fig. SF4 for S7, supplementary Fig. SF5 for S8, supplementary Fig. SF6 for S9, supplementary Fig. SF7 for S10, supplementary Fig. SF8 for S11, supplementary Fig. SF9 for S12, supplementary Fig. SF10 for S13, supplementary Fig. SF11 for S14, and supplementary Fig. SF12 for S15.

1.1 Detailed analysis of the results per subject

Subject S1: The pre-surgical icEEG analysis determined the plausible epileptogenic zone (EZ) at the right hippocampus. sLORETA, MSP and cMEM methods showed good accuracy in localization. Fig. 3 shows that the MSP solution is closer to the hippocampus than the sLORETA and cMEM solutions.

Subject S2: The pre-surgical icEEG analysis determined the plausible EZ at the left basal temporal lobe. All methods showed good accuracy in localization with respect to the VOI (volume of interest). However, the solution obtained with MSP is more focal and has slightly better accuracy the than the sLORETA and cMEM solutions.

Subject S3: The pre-surgical analysis determined the plausible EZ at the left medial frontal lobe. The three methods showed good accuracy in localization. The sLORETA solution that better described the scalp EEG has a medial positive and an upper negative source, whereas the best MSP and cMEM solutions only contain medial positive sources and they coincide. The reconstructed EEG with the MSP and cMEM solutions are more similar to the EEG than the sLORETA solution.

Subject S4: The pre-surgical icEEG analysis determined the plausible EZ at the central sulcus and surrounding area. The three methods showed bad localization. However, the sources with strongest activity with reversed polarity (in blue) in the sLORETA and cMEM solutions are located very close to the actual VOI, which is the smallest among all subjects. The white/gray matter segmentation from the MRI (magnetic resonance image) of this subject was particularly difficult due to the age of this subject and the structural abnormality around the resected region.

Subject S5: The pre-surgical analysis determined the plausible EZ in the left anterior temporal lobe. The three methods showed very good accuracy with very similar solutions. Note that the MSP solution is more focal than sLORETA and cMEM solutions.

Subject S6: The pre-surgical icEEG analysis determined the plausible EZ at the right ventromedial prefrontal lobe. The three methods showed very good accuracy with a more focal solution given by the MSP and the cMEM solution is slightly more anterior than sLORETA and MSP solutions.

Subject S7: The pre-surgical MRI showed a left medial frontal focal cortical dysplasia. The three methods showed good accuracy, although they do not match exactly. The MSP inverse polarity peaks (in blue) are more medial than the sLORETA and cMEM solutions. The sLORETA solution has one negative medial and one positive cortical peak that make the synthetic EEG look more similar to the measurements than the synthetic EEG generated with the MSP and cMEM solutions.

Subject S8: The pre-surgical MRI showed a left hippocampal sclerosis and the resected region was in the left anterior temporal lobe. The three methods fail to localize the VOI although the MSP is much closer than the other two. The MSP solution presents maximum activity at the left ventromedial prefrontal lobe. This region is difficult to distinguish from the VOI because they have a similar normal orientation. Note that both sLORETA and cMEM have a secondary positive peak at the same location of the MSP main positive peak. We believe that slight differences in the ways the sources are weighted by each algorithm make both sLORETA and cMEM to fail.

Subject S9: The pre-surgical PET showed a right posterior temporal hypometabolism. The three methods were not able to localize the VOI, although the cMEM is much closer than the other two. Note that both sLORETA and, more notoriously MSP, have a secondary negative peak at the same location of the cMEM main negative peak. We believe that slight differences in the ways the sources are weighted by each algorithm make both sLORETA and MSP to fail.

Subject S10: The pre-surgical icEEG analysis determined the plausible EZ to be in the right medial temporal lobe and pole. The three methods showed good accuracy, although the sLORETA solution is positive and medial and the MSP and cMEM solutions are negative and more basal.

Subject S11: The pre-surgical MRI showed a right medial temporal lobe lesion. The three methods showed good accuracy, although they do not coincide in a unique spot. The sLORETA solution is not fully inside the VOI (see Table 2) due to a ventral frontal lobe maximum. The MSP solution is more clearly located in the temporal region and more anterior than the sLORETA solution whereas the cMEM solution is much more posterior but still inside the VOI.

Subject S12: The pre-surgical MRI showed a left medial temporal and left frontal lobe lesion, and PET showed a left temporal lesion. sLORETA and MSP showed good accuracy and the solution maximum correspond to a more medial source for both methods. On the contrary, cMEM localizes the strongest activity at the left ventral prefrontal lobe, outside the VOI. This region is difficult to distinguish from the VOI because they have a similar normal orientation.

Subject S13: The pre-surgical MRI showed a right frontal lesion. The three methods showed good accuracy and convergence in showing inward-flowing activity in the frontal cortex and outward-flowing activity in the medial wall of the right hemisphere. However, the MSP and cMEM solutions are more intense at the top part of the cortex whereas the sLORETA solution is more intense at the medial wall. Note that the resected VOI is the largest among all subjects.

Subject S14: The pre-surgical MRI showed a left parahippocampal sclerosis and the resection included part of the left anterior-medial temporal lobe. sLORETA and MSP showed good accuracy with respect to the VOI. On the contrary, cMEM localizes the strongest activity at the left ventral prefrontal lobe, outside the VOI, although the VOI touches the same Brodmann area (BA11).

Subject S15: The pre-surgical MRI was normal, and the PET showed a left anterior temporal hypometabolism. The three methods showed good accuracy with respect to the VOI and a very similar activation map.
