# Supplementary material for: Source localization of epileptic spikes using Multiple Sparse Priors
Source: Clin Neurophysiol. Author manuscript; Available in PMC 2021 Mar 18. (PMC7971150; doi:10.1016/j.clinph.2020.10.030)
Supplement: Appendices A and B Final [file NIHMS1666397-supplement-Appendices_A_and_B_Final.pdf]

Source localization of epileptic spikes using Multiple Sparse Priors

Mariano Fernandez-Corazza[1*#], Rui Feng[2#], Chengxin Ma[2], Jie Hu[2], Li Pan[2], Phan Luu[3,4] and Don Tucker[3,4]

[1]LEICI Instituto de Investigaciones en Electrónica, Control y Procesamiento de Señales, Universidad Nacional de La Plata - CONICET, Argentina

[2]Department of Neurosurgery, Huashan Hospital of Fudan University, Shanghai, China

[3]Brain Electrophysiology Laboratory (BEL) Company, Eugene, OR, USA

[4]NeuroInformatics Center, University of Oregon, Eugene, OR, USA

[#] These authors contributed equally to the work presented in this paper.

* Correspondence: Mariano Fernández-Corazza, LEICI Instituto de Investigaciones en Electrónica, Control y Procesamiento de Señales, Facultad de Ingeniería, Universidad Nacional de La Plata, CC91 (1900), La Plata, Buenos Aires, Argentina.

E-mail: marianof.corazza@ing.unlp.edu.ar

1 Appendix A: hexahedral FEM validation

In this work we used a hexahedral finite element model (HexaFEM) to compute the forward problem of EEG. In fact, we computed the transcranial electrical stimulation (TES) forward problem which consists of applying an electric current on the scalp. Then, the EEG lead field matrix is obtained from the TES solutions by using the reciprocity principle. In this appendix, we show the validation against the analytic solutions in a spherical model and against the finite difference method (FDM) solver of Geo Source 3 Philips system in a realistic head model. The analytic formulation can be found elsewhere (Fernández-Corazza et al., 2011).

1.1 Comparison on a three-layer sphere

We built a three-layer sphere with radii of 9.2, 8.5 and 7.5 centimeters and conductivities 0.33, 0.01 and 0.33 S/m respectively. The electrode layout used is the spherical 256-channel Geodesic sensor-net, with electrode 257 being the reference Cz. A unitary electrical current source was applied to each electrode pair with Cz being fixed. Approximately 2500 dipoles were placed at the internal compartment (separated approximately 10mm from the layer) with normal orientation pointing outwards.

Fig. A.1 shows examples of the lead fields for comparison. The normalized relative difference measure (NRDM) (Mejis et al 1989) and the magnitude error (MAG) metrics were computed to

quantify the differences. The NRDM is a metric that compares two vectors of the same size based only on the shape of them and not on their strength: it is zero if vectors are colinear, the squared root of 2 if the vectors are orthogonal, and 2 if the vectors are colinear but opposite. The MAG metric compares two vectors of the same size based only on their magnitude: it is 1 if both vectors have equal ℓ_2-norm, < 1 if the ℓ_2-norm of the first vector is lower than the ℓ_2-norm of the second vector, and >1 vice versa.

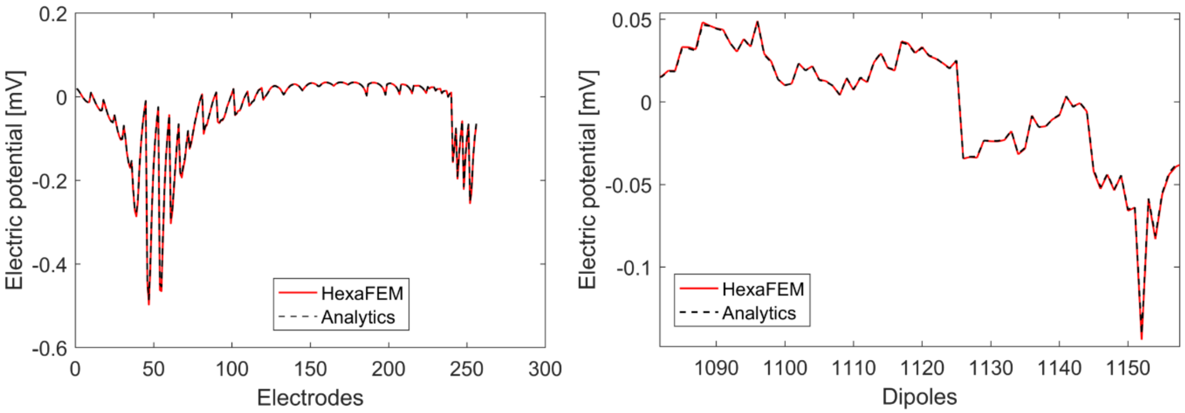

Figure A.1: Lead field matrix comparison. Left: potential at the electrodes generated by one dipole (as an example) computed by analytics and HexaFEM. The comparison metrics between HexaFEM and analytics are NRDM=0.013 and MAG=1.02. Right: electric potential at electrode 1 (example) as a function of the oriented dipolar sources (zoomed). The comparison metrics between HexaFEM and analytics are NRDM=0.011 and MAG=1.02.

1.2 Comparison on a realistic head model.

Similarly to the three layer sphere, we compared the HexaFEM and FDM solutions for one realistic head model using the same conductivity values. Fig. A.2 shows this comparison.

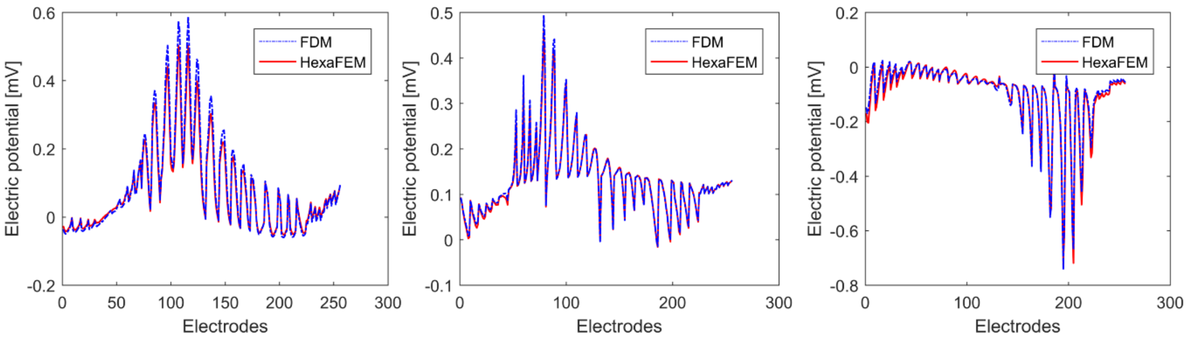

Figure A.2: Examples of the EEG lead field matrix for three different dipoles (first, intermediate, and last dipoles) computed with HexaFEM and FDM. The resulting metrics are: NRDM=0.048, NRDM=0.016, and NRDM=0.09; and MAG=1.13, MAG=1.12, and MAG=0.96 respectively for the three dipoles.

2 Appendix B: Details of MSP

The typical signal model $\mathbf{Y}$ for L electrodes, D dipoles and a time window with T samples is:

$$\mathbf{Y}_{L\times T} = \mathbf{L}_{L\times D} \cdot \mathbf{\Theta}_{D\times T} + \mathbf{X} \cdot \boldsymbol{\beta} + \mathbf{N}_{L\times T}, \qquad (B.1)$$

where $\mathbf{L}$ is the lead field matrix, $\mathbf{\Theta}$ are the sources, $\mathbf{X} \cdot \boldsymbol{\beta}$ accounts for "fixed effects" or "confounds" and $\mathbf{N}$ is the noise matrix. $\mathbf{\Theta}$ and $\mathbf{N}$ are modeled as matrix normal random variables:

$$\mathbf{\Theta} \sim \mathcal{N}(0, \mathbf{V}, \mathbf{\Sigma}^{\epsilon});\ \mathbf{N} \sim \mathcal{N}(0, \mathbf{V}, \mathbf{\Sigma}^{n}), \qquad (B.2)$$

where $\mathbf{V}$ is the temporal covariance matrix and $\mathbf{\Sigma}$ is the spatial covariance matrix. Note that the sample covariance estimators are: $\widehat{\mathbf{V}} \approx \mathbf{Y}^T\mathbf{Y}$, and $\widehat{\mathbf{\Sigma}} \approx \mathbf{Y}\mathbf{Y}^T$.

The first step is to project the data to eliminate the $\mathbf{X} \cdot \boldsymbol{\beta}$ term (model reduction). This is typically done assuming one confound that accounts for the constant shift of the potential reference: $\mathbf{X}_{L\times 1} = ones(L, 1)$. Thus, the spatial projector is:

$$\mathbf{U}_{L\times L} = (\mathbf{I} - \mathbf{X}\mathbf{X}^{-}) \qquad (B.3)$$

There is also a spatial projection matrix $\mathbf{S}$ that is built as described in Friston et al. (2008) as the principal eigenvectors of the sample covariance matrix over time. After the projection:

$$\widetilde{\mathbf{Y}} = \widetilde{\mathbf{L}} \cdot \widetilde{\mathbf{\Theta}} + \widetilde{\mathbf{N}};\ \widetilde{\mathbf{\Theta}} \sim \mathcal{N}\left(0, \widetilde{\mathbf{V}}, \mathbf{\Sigma}^{\epsilon}\right);\ \widetilde{\mathbf{N}} \sim \mathcal{N}\left(0, \widetilde{\mathbf{V}}, \widetilde{\mathbf{\Sigma}^{n}}\right), \qquad (B.4)$$

where $\widetilde{\mathbf{V}}$ and $\widetilde{\mathbf{\Sigma}^{n}}$ are the projected covariance matrices in the signal space, $\widetilde{\mathbf{L}} = \mathbf{U}^T\mathbf{L}$, $\widetilde{\mathbf{\Theta}} = \mathbf{\Theta}\mathbf{S}$ and $\widetilde{\mathbf{Y}} = \mathbf{U}^T\mathbf{Y}\mathbf{S}$. $\mathbf{\Sigma}^{\epsilon} = \sum_{i=1}^{m} \exp(\lambda_i)\, \mathbf{Q}^i$, where $\mathbf{Q}^i$ are different empirical source covariance priors with unknown hyperparameters λ_i. They are typically "sparse" priors such as some small subnetworks, or patches. As $\mathbf{\Sigma}^{\epsilon}$ is in the source space, there is no projection needed. Lastly, $\mathbf{\Sigma}^{n} = \lambda_n\mathbf{I}$ models uncorrelated sensor noise.

Both noise and source models are merged into one stochastic model by performing a projection to the signal space:

$$\widetilde{\mathbf{Y}} = \widetilde{\mathbf{L}} \cdot \widetilde{\mathbf{\Theta}} + \widetilde{\mathbf{N}} \qquad (B.5)$$

$$\widetilde{\mathbf{Y}} \sim \mathcal{N}\left(0, \widetilde{\mathbf{V}}, \widetilde{\mathbf{L}}\mathbf{\Sigma}^{\epsilon}\widetilde{\mathbf{L}}^T + \widetilde{\mathbf{\Sigma}^{n}}\right) \qquad (B.6)$$

The second step is the estimation of the hyperparameters (λ_i) from data (or "evidence"). For this purpose, Friston et al. (2008) propose two methods: "greedy search" and automatic relevance determination (ARD). The latter is sparse in nature and it starts with a maximum number of non-zero hyperparameters and it attempts to reduce them. The ARD approach is the one used in both Friston et al. (2008) and here. The hyperparameters λ_i are estimated with an expectation-maximization method, where the M-step estimates the λ_i hyperparameters and the E-step computes the expected value of the $\mathbf{\Theta}$ estimates.

Once the hyperparameters corresponding to each prior are computed using the expectation-maximization method, the spatial covariance matrix in the source space is built as:

$$\boldsymbol{\Sigma}^{\epsilon}_{Source\ Space} = \sum_{i=1}^{m} h(i+1)\mathbf{Q}_i \qquad (B.7)$$

And then projected to the signal space by doing:

$$\boldsymbol{\Sigma}_{\text{Electrode space } (L\times L)} = h(1)\mathbf{U}^T\mathbf{C}_{nn}\mathbf{U} + \sum_{i=1}^{m} h(i+1)\tilde{\mathbf{L}}\,\mathbf{Q}_i\,\tilde{\mathbf{L}}^T \qquad (B.8)$$
